# Population genomics reveals that an anthropophilic population of *Aedes aegypti* mosquitoes in West Africa recently gave rise to American and Asian populations of this major disease vector

**DOI:** 10.1186/s12915-017-0351-0

**Published:** 2017-02-28

**Authors:** Jacob E. Crawford, Joel M. Alves, William J. Palmer, Jonathan P. Day, Massamba Sylla, Ranjan Ramasamy, Sinnathamby N. Surendran, William C. Black, Arnab Pain, Francis M. Jiggins

**Affiliations:** 1 Department of Integrative Biology, University of California, Berkeley, CA 94720-3140 USA; 2 Present Address: Verily Life Sciences, South San Francisco, CA 94080 USA; 3 Department of Genetics, University of Cambridge, Downing Street, Cambridge, CB2 3EH UK; 4 CIBIO/InBIO, Centro de Investigação em Biodiversidade e Recursos Genéticos, Campus Agrário de Vairão, Universidade do Porto, 4485-661 Vairão, Portugal; 5 Department of Microbiology, Immunology and Pathology, Colorado State University, Fort Collins, CO USA; 6 ID-FISH Technology, Palo Alto, CA 94303 USA; 7 Department of Zoology, University of Jaffna, Jaffna, Sri Lanka; 8 Biological and Environmental Sciences and Engineering Division, KAUST, Thuwal, Kingdom of Saudi Arabia

**Keywords:** *Aedes aegypti*, Anthropophilic, Dengue virus, Zika virus, Arboviral diseases, Mosquito evolution, Vector-borne diseases

## Abstract

**Background:**

The mosquito *Aedes aegypti* is the main vector of dengue, Zika, chikungunya and yellow fever viruses. This major disease vector is thought to have arisen when the African subspecies *Ae. aegypti formosus* evolved from being zoophilic and living in forest habitats into a form that specialises on humans and resides near human population centres. The resulting domestic subspecies, *Ae. aegypti aegypti*, is found throughout the tropics and largely blood-feeds on humans.

**Results:**

To understand this transition, we have sequenced the exomes of mosquitoes collected from five populations from around the world. We found that *Ae. aegypti* specimens from an urban population in Senegal in West Africa were more closely related to populations in Mexico and Sri Lanka than they were to a nearby forest population. We estimate that the populations in Senegal and Mexico split just a few hundred years ago, and we found no evidence of *Ae. aegypti aegypti* mosquitoes migrating back to Africa from elsewhere in the tropics. The out-of-Africa migration was accompanied by a dramatic reduction in effective population size, resulting in a loss of genetic diversity and rare genetic variants.

**Conclusions:**

We conclude that a domestic population of *Ae. aegypti* in Senegal and domestic populations on other continents are more closely related to each other than to other African populations. This suggests that an ancestral population of *Ae. aegypti* evolved to become a human specialist in Africa, giving rise to the subspecies *Ae. aegypti aegypti.* The descendants of this population are still found in West Africa today, and the rest of the world was colonised when mosquitoes from this population migrated out of Africa. This is the first report of an African population of *Ae. aegypti aegypti* mosquitoes that is closely related to Asian and American populations. As the two subspecies differ in their ability to vector disease, their existence side by side in West Africa may have important implications for disease transmission.

**Electronic supplementary material:**

The online version of this article (doi:10.1186/s12915-017-0351-0) contains supplementary material, which is available to authorized users.

## Background

Arthropod-borne viruses (arboviruses) are a major threat to human health in many tropical and subtropical countries. The most important vector of human arboviruses is the mosquito *Aedes aegypti,* which transmits dengue, chikungunya, yellow fever and Zika viruses. A widespread epidemic of the Zika virus has recently occurred across South America, Central America and the Caribbean and has been linked to fetal brain abnormalities [1]. Over the last decade, chikungunya virus, which is transmitted by both *Aedes albopictus* and *Ae. aegypti,* has emerged as a major cause for concern, causing epidemics in Asia and many Indian Ocean islands as well as in southern Europe and the Americas [2]. Dengue virus, which is responsible for the most common human arboviral disease infecting millions of people every year, has greatly increased its range in tropical and subtropical regions [3, 4].

*Ae. aegypti* occurs throughout the tropics and subtropics, but populations vary in their ability to transmit disease (vector capacity) [5–11]. Outside of Africa, *Ae. aegypti* has a strong genetic preference for entering houses to blood-feed on humans and an ability to survive and oviposit in relatively clean water in man-made containers in the human environment [5, 6]. However, across sub-Saharan Africa there is considerable variation among populations in their ecology, behaviour and appearance [10, 12–15]. Some populations are less strongly human associated, being found in forests, ovipositing in tree holes and feeding on other mammals [5–8]. Elsewhere, populations have become ‘domesticated,’ developing in water in and around homes and feeding on humans. Aside from a few locations on the coast of Kenya that appear to have been colonised by non-African populations, African populations tend to cluster together genetically regardless of whether they are forest or domestic forms [12]. This was interpreted as suggesting that these human-associated populations in Africa have arisen independently from the domestic populations found elsewhere in the tropics [12]. However, as we discuss later, such interpretations of genetic data can be misleading.

*Ae. aegypti* has long been hypothesised to have originated in Africa, probably travelling in ships along trading routes [7, 8]. This out-of-Africa model has been supported by genetic data, as African populations have higher genetic diversity than those from elsewhere in the tropics [16]. Furthermore, rooted trees constructed from the sequences of a small number of nuclear genes have consistently found that the genetic diversity in Asian and New World populations is a subset of that found in Africa [16]. The exact origin of this migration out of Africa remains uncertain. Furthermore, it is not known whether the species evolved to specialise on humans in Africa or after it had migrated out of Africa [17].

The species *Ae. aegypti* has been split into two subspecies [7]. Outside Africa, nearly all populations belong to the subspecies *Ae. aegypti aegypti,* which is light in colour and strongly anthropophilic. In Africa the subspecies *Ae. aegypti formosus* is darker in colour and lives in forested habitats. The two subspecies were originally defined based on these differences in colouration, with *Ae. aegypti aegypti* having pale scales on the first abdominal tergite [7]. However, West African populations that have these pale scales appear to be genetically more similar to *Ae. aegypti formosus* populations than *Ae. aegypti aegypti* from elsewhere in the tropics [10, 14, 15]*.* This has led some authors to call all African populations *Ae. aegypti formosus,* while others have continued to use the original morphological definition.

Population genetics studies of *Ae. aegypti* have a long history, but until recently they were limited by the small numbers of genetic markers available. Whole genome sequencing is prohibitively expensive due to the large genome size [18], but three approaches have made genome-scale analyses possible. Restriction site-associated DNA (RAD) sequencing has been used to score large numbers of single nucleotide polymorphisms (SNPs) [16, 19, 20], although the repetitive genome coupled with PCR duplicates due to the low DNA yield of mosquitoes can complicate this approach [20]. An *Ae. aegypti* SNP chip can genotype more than 25,000 SNPs [21], although the analysis of these data can be complicated because a biased set of SNPs is genotyped [22]. Finally, we recently developed exome capture probes, which allow the protein-coding regions of the genome to be selectively resequenced [23]. This makes sequencing affordable, minimises ascertainment bias and avoids repetitive regions where it is difficult to map short sequence reads.

Here we have used exome sequencing to investigate the origins of the domestic *Ae. aegypti aegypti* populations that are the main vectors of human viruses. To do this, we sampled mosquitoes from two nearby populations in Senegal, West Africa, one of which was from a forested region and has the classical phenotype of *Ae. aegypti formosus,* and the other of which was from an urban location and resembled *Ae. aegypti aegypti.* These samples were then compared to populations from East Africa, Mexico and Sri Lanka. We found that the domestic population in West Africa is most closely related to domestic populations in Mexico and Sri Lanka. We conclude that the species likely became domesticated in Africa, and the migration out of Africa came from populations related to extant domestic African populations. Furthermore, the out-of-Africa migration and probably the original domestication event in Africa were associated with population bottlenecks.

## Methods

### Mosquito samples

We investigated *Ae. aegypti* from five populations (the sample details are given in Additional file 1). Wherever possible, mosquitoes were sampled from multiple nearby sites. Mexican mosquitoes were all collected from independent sites in Yucatán state and supplied as extracted DNA by William Black. This group of mosquitoes was a mixture of males and females, with the sex of individuals unknown. The collection sites were urban and peri-urban. Female Sri Lankan *Ae. aegypti* were supplied by Ranjan Ramasamy and Sinnathamby Surendran. Nine individuals from the Jaffna district [24] and one from the Batticaloa district [24] had been collected from separate oviposition traps in 2012 and reared to adulthood in the laboratory. These specimens were from urban and peri-urban areas. Female Ugandan *Ae. aegypti* were supplied by Jeff Powell. They had been collected in Lunyo, Entebbe in 2012 using oviposition traps and reared in the laboratory.

The samples from two populations in Senegal were supplied as extracted DNA by William Black [10]. They fell into two phenotypically and geographically distinct groups. The first of these we called ‘Senegal Forest’; this group is from the rural forested locations near Kedougou [10]. Here the mosquitoes lacked pale scales on the first abdominal tergite, which is the classical phenotype associated with *Ae. aegypti formosus* [10, 25]. This group of mosquitoes was a mixture of males and females, with the sex of individuals unknown. The second group of mosquitoes, which we call ‘Senegal Urban’, came from the urban location of Kaolack and had the pale scales on the first abdominal tergite that are classically associated with *Ae. aegypti aegypti* [10, 25]*.* This sample consisted of 2 males and 10 females. The two locations are approximately 420 km apart.

*Aedes bromeliae* eggs were collected in July 2010 from Kilifi in coastal Kenya using oviposition traps. Eggs were hatched in the laboratory in the UK and reared to maturity. A single female was then used for sequencing.

#### Library preparation and sequencing

DNA was extracted from *Ae. aegypti* mosquitoes using the DNeasy Blood and Tissue Kit (Qiagen). Illumina sequencing libraries were constructed from individual mosquitoes using the Illumina TruSeq Library Prep Kit. The concentration of each library was estimated by quantitative PCR, and four equimolar pools of the libraries from Mexico, Senegal, Uganda and Sri Lanka were made. Exome capture was then performed to enrich for coding sequences using custom SeqCap EZ Developer probes (Nimblegen) [23]. Overlapping probes covering the protein-coding sequence, not including untranslated regions (UTRs), in the AaegL1.3 gene annotations [18] were produced by Nimblegen based on coding sequence coordinates (covering 22.2 Mb) specified by us. In total, 26.7 Mb representing 2% of the genome was targeted by capture probes, which includes regions flanking the coding sequence that were added during the proprietary design process. Exome capture coordinates are available in Additional file 2 (from [23]). Each of the four exome-captured pools of libraries was then separately sequenced in one lane each of 100-bp paired-end HiSeq2000 runs by the Beijing Genomics Institute (China).

DNA was then extracted from a single *Ae. bromeliae* individual using the QIAamp DNA Mini Kit. A whole-genome sequencing library was constructed using the Illumina Nextera DNA Library Prep Kit. This library was sequenced in one lane of MiSeq (2 × 250 bp paired-end reads; Oxford Genomics) and two lanes of HiSeq2000 (2 × 100 bp paired-end reads; King Abdullah University of Science and Technology, KAUST, sequencing core).

#### Sequence alignment and variant calling

Initially *Aedes aegypti* reads were demultiplexed using fastq-grep [26] and hard matching of Illumina barcodes. As such, reads with any errors in barcode sequence were discarded. The following steps were then performed on reads from each of the populations, and *Aedes bromeliae*, separately.

Paired reads were quality trimmed from the 3′ end, cutting when average quality scores in sliding windows of 5 bp dropped below 30, and trimmed when the quality score at the end of the read dropped below 30 using Trimmomatic version 0.27 [27]. As the insert size from some individuals was shorter than the length of two sequencing reads, we initially observed some sequence overlap of paired-end reads. This is undesirable, as when mapped they violate the later sampling assumption that a given SNP observation results from a single molecule. As such, overlapping reads were merged into single pseudoreads with FLASH version 1.2.11 [28] and then treated as single-end sequencing reads. Both paired- and single-end pseudoreads were then aligned to the *Aedes aegypti* reference genome AaegL3.3 using BWA-MEM version 0.7.10 [29]. Unmapped reads as well as those mapping below a mapQ of 30 were then discarded using SAMtools view [30]. SAMtools was then used to merge and sort the paired- and single-end pseudoreads read alignments into a single BAM file, which was used for all subsequent analyses. We observed a number of *Ae. bromeliae* reads mapping with coordinates outside the normal range, so for this set we used a custom script to remove read pairs with mapping start positions less than 100 bp or greater than 400 bp. Reads were then realigned around indels using GATK version 3.4-0 [31], and both optical and PCR duplicates were removed using Picard [32] version 1.90. An uncompressed BCF was generated using SAMtools mpileup version 0.1.19 with Indel calling disabled; skipping bases with a baseQ/BAQ less than 30; and mapQ adjustment (-C) set to 30. This was finally converted to a VCF using bcftools. Low-quality SNPs were removed by using SNPcleaner version 2.2.4 [33] to remove sites that had a total depth across all individuals of >1500 or had less than 10 individuals with at least 10 reads. Additional sites were filtered based on default settings within the SNPcleaner script. VCF files were queried using SNPcleaner for each population separately in order to obtain a set of robust sites for analysis. This list was used as a -sites file input for ANGSD [34], such that subsequent analysis within ANGSD was restricted to these sites. For some analyses that require comparison among populations, we found the intersect between the lists of high-quality sites for each population and used this common set for analysis. Minimum map quality and base quality thresholds of 30 and 20 were used. For some analyses we converted genotype likelihoods into hard-called genotypes using the doGeno function in ANGSD with a cutoff of 0.95 for posterior probabilities on the genotype calls and a minimum read depth of 8. This read processing and genotype calling process was repeated for the sequence reads from *Ae. bromeliae*, except that the *Ae. aegypti* sites list was used since SNPcleaner is not intended for single diploid samples*.*

#### Population genetics analysis

We estimated the nucleotide diversity π using ANGSD, which calculates π based on estimates of per-site allele frequencies across each population sample (i.e. without the need to call genotypes), directly accounting for sample size and read depth. We estimated 95% bootstrap confidence intervals (CIs) by resampling scaffolds with replacement 500 times and recalculating the statistic. As nucleotide diversity is reduced in coding sequence due to purifying selection, we only used sites >500 bp from exons in this analysis (≥399,259 in each population).

To construct a neighbour-joining tree of our samples, we first estimated the pairwise genetic distance (*D*_*xy*_) between all pairs of samples based on genotype calls. *D*_*xy*_ was calculated from the called genotypes as (*h* + 2*H*)/2 *L*, where *h* is the number of sites where one or both individuals carry heterozygous genotypes, *H* is the number of sites where the two individuals are homozygous for different alleles and *L* is the number of sites where both individuals have called genotypes.

To investigate population structure and the ancestry of individual mosquitoes, we performed an admixture analysis using NGSadmix, which makes inferences based on genotype likelihoods [35]. We also analysed data from the three chromosomes separately using the chromosome assignments of Juneja et al. [20]. As an alternative approach to investigate genetic structure, we performed a principal component analysis (PCA). The PCA was based on a covariance matrix among individuals that was computed while accounting for genotype uncertainty using the function ngsCovar in ngsTools [33].

We calculated *F*_*ST*_ [36] between populations from allele frequencies estimated for each population directly from read data using ANGSD. This analysis used data from 17,351,731 coding and non-coding sites with no minimum minor allele frequency.

We investigated the historical relationships between our populations by reconstructing a population maximum likelihood tree based in allele frequencies using the program TreeMix [37]. This analysis used all high-quality coding and non-coding sites in our dataset, and *Ae. bromeliae* was used as an outgroup. We chose this species, as the more closely related outgroup *Ae. mascarensis* frequently shares polymorphisms with *Ae. aegypti* [16]*.* To account for the non-independence of sites due to linkage disequilibrium, we used a block size (*k*) of 100 SNPs. To evaluate the confidence in the inferred tree topology, 1000 bootstrap replicates were conducted by resampling blocks of 100 SNPs. To test whether there had been migrations between the populations after they split, we used the three- and four-population tests of Reich et al. [38], also implemented in TreeMix.

We estimated one- and two-dimensional site frequency spectra (SFS) using the doSaf function within ANGSD to estimate per-site allele frequencies combined with the realSFS program [39] to optimize the genome-wide SFS. We minimised the effect of natural selection on the SFS by including only third codon position sites as well as non-coding sites more than 100 bp from the nearest exon, and as before, only sites passing all filters were included for analysis. Approximately 6.44 Mb was included in this dataset. To facilitate comparison among populations, we down-sampled the larger population samples and chose 10 randomly selected individuals from each population. Two-dimensional (2D) spectra were plotted using *dadi* [40]*.*

We fit two classes of demographic models to the data from Senegal Forest, Senegal Urban and Mexico using fastsimcoal2 version 2.5.2 [41] to distinguish between the hypotheses that Senegal Urban is evolutionarily intermediate because it (1) is admixed with domesticated, non-African ancestry, or (2) represents the domesticated form within Africa that is the genetic ancestor of non-African domesticated populations. We first fit simple three-population models with no size changes for each of the two classes, and then fit a second version of the model including size changes in each of the three populations. Schematics of the two models and their parameters can be found in Additional file 3.

We note that for the admixture models, the order of divergence times for Mexico and Senegal Urban was not specified such that either could diverge before the other from Senegal Forest. In addition, we fixed the current effective size of Senegal Forest to 1,000,000 in order to anchor the models and reduce the number of free variables. To obtain best-fit parameter values, we first conducted a round of 500 optimizations for each model using wide parameter ranges and the following fastsimcoal2 parameters: -n 1000 -N 100000 -c0 -d -M 0.001 -l 10 -L 40. Simulations were structured to model exomes by simulating 17,000 independent regions using the mutation rate estimated for *Drosophila melanogaster*, 3.5 × 10^–9^ [42], since this parameter is not available for mosquitoes, and an equivalent within-region recombination rate. We then conducted a second round of 500 optimizations using a more narrow set of possible starting parameter values tuned on the first set of optimizations in order to improve model fitting. We used the parameter values from the replicate with the highest likelihood value from the second set of optimizations as the best-fit model and used this model for a final likelihood calculation by conducting a final set of 10^6^ simulations for a more accurate calculation of the likelihood value. Confidence values were estimated for model parameters using block-bootstrapping, where 100 bootstrapped datasets were generated by arbitrarily assembling scaffolds into a contiguous pseudochromosome, dividing this ‘chromosome’ into 1000 identically sized blocks and resampling with replacement. Best-fit models were obtained for each bootstrapped dataset using a set of 50 optimizations with broad starting parameter value ranges. The same bootstrapping approach was performed to obtain 95% CIs for 1D site frequency spectra as well.

We scanned the exome for regions with exceptional genetic differentiation consistent with the action of recent positive selection using a normalised version of the population branch statistic (*PBSn1*) [43]:

$$ PB{S}_{n1}=\frac{PB{S}_1}{1+ PB{S}_1+ PB{S}_2+ PB{S}_3} $$

where *PBS*_*1*_ indicates *PBS* calculated with the domesticated population as the focal population, *PBS*_*2*_ indicates *PBS* calculated with the Ugandan population as the focal population and *PBS*_*3*_ indicates *PBS* calculated with Senegal Forest as the focal population. For this analysis, we obtained admixture-corrected allele frequencies using NGSadmix analysis but with no minimum allele frequency filter. We then used allele frequencies to calculate *F*_*ST*_ between the focal population (Sri Lanka, Senegal Urban or Mexico) and both Senegal Forest and Uganda. These values were then used to calculate *PBSn1* for non-overlapping blocks of 5 SNPs. We annotated top windows by identifying the gene (*Ae. aegypti,* AaegL3.3) with the exon on or nearest the most differentiated SNP within the window and pulling external metadata for these genes from VectorBase [44].

For each population pairwise comparison we calculated the Weir and Cockerham *F*_*ST*_ at each variant position (using the hard-called genotypes generated from ANGSD) with VCFtools version 0.1.12 [45]. All positions with less than 10 individuals in each population comparison were excluded. The annotation for each candidate SNP was determined using SnpEff, version 4.1 [46].

Final plots were generated in R [47] using the built-in functions and the R package ggplot2 [48].

## Results

### High-coverage population exome sequences and an *Ae. bromeliae* genome sequence

The *Ae. aegypti* genome is large (1.4 GB), repetitive and poorly assembled, which makes it expensive and challenging to resequence [18, 23]. To overcome this, we used probes to capture the predicted protein-coding sequence [23], which both reduces the cost of sequencing and avoids the repetitive and most poorly assembled regions of the genome. In total, we sequenced 15 mosquitoes from Uganda, 22 from Senegal, 10 from Sri Lanka and 24 from Mexico. Each mosquito was individually barcoded in the sequencing library. The exome capture was efficient, with 89% of mapped reads on target, resulting in >400X greater coverage of the exome compared to the genome average. The mean on-target coverage of the exomes was 29X, with the mean coverage of individual mosquitoes ranging from 15X to 48X. In total we genotyped 17,351,731 sites, 1,321,924 of which were variable when genotypes were called. We called 436,559 polymorphisms in Mexico, 782,744 in Senegal Forest, 464,665 in Senegal Urban, 286,307 in Sri Lanka and 645,547 in Uganda.

For many types of analyses it is helpful to have the genome sequence of a relatively closely related species as an outgroup. For this reason we sequenced the whole genome of *Ae. bromeliae* and mapped the reads to the *Ae. aegypti* reference genome*.* In total we called genotypes at 104,017,808 sites*.* Of the 17,351,731 sequenced sites in the *Ae. aegypti* dataset, 13,806,549 (80%) had called genotypes in *Ae. bromeliae.* The mean coverage of the exome was 6.54X; coverage of intergenic regions was substantially lower (presumably due to low rates of mapping).

### Reduced genetic diversity and fewer rare variants support the out-of-Africa migration of *Ae. aegypti*

**Fig. 1:**
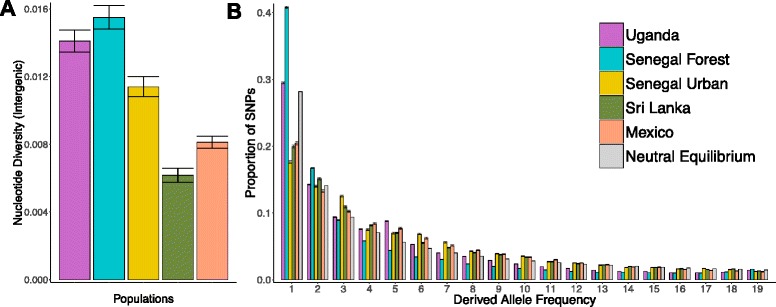
Nucleotide diversity (**a**) and site frequency spectrum (**b**) of five populations of *Ae. aegypti.* **a** Nucleotide diversity (π) was estimated for non-coding sites >500 bp from exons. **b** The site frequency spectrum was estimated for 10 individuals from each population using third codon positions and non-coding sites >100 bp from exons. *Ae. bromeliae* was used to polarize sites. The *grey bars* are the expected frequencies assuming variant sites are neutral and the effective population size is constant. In both panels, error bars are 95% confidence intervals from block-bootstrapping

*Ae. aegypti* is believed to have originated in Africa and subsequently colonised Asia and the Americas [7, 8, 12]. We found that the genetic diversity (π) of our three African populations was substantially higher than those from Mexico and Sri Lanka, which is consistent with a population bottleneck during the out-of-Africa migration (Fig. 1a). Interestingly, our domestic population from West Africa (Senegal Urban) has a nucleotide diversity that is intermediate between the other African populations and those from outside Africa (Fig. 1a). This indicates that historically the effective population size of this population has been reduced below that of the nearby Senegal Forest population.

Population bottlenecks and other changes in the effective population size not only alter the nucleotide diversity but also the allele frequency spectrum [49]. There has been a striking reduction in the number of rare alleles in the Mexican and Sri Lankan populations relative to both the neutral, equilibrium expectation and the populations in Uganda and Senegal Forest (Fig. 1b). This loss of rare variants is expected if these populations have experienced a population bottleneck [50]. Unexpectedly, the domestic Senegal Urban population has a similar reduction in rare variants, suggesting that it too may have experienced a population bottleneck in its history (Fig. 1b). Interestingly, the Senegal Forest population has an excess of rare variants compared to the neutral expectation. This may indicate a recent increase in population size in this population, but it could also reflect the fact that a large proportion of our data is protein-coding sequences, and it is common to find that purifying selection keeps slightly deleterious amino acid polymorphisms at a low frequency [51].

### Anthropophilic *Ae. aegypti* from Senegal are genetically distinct from other African populations and populations outside of Africa

**Fig. 2:**
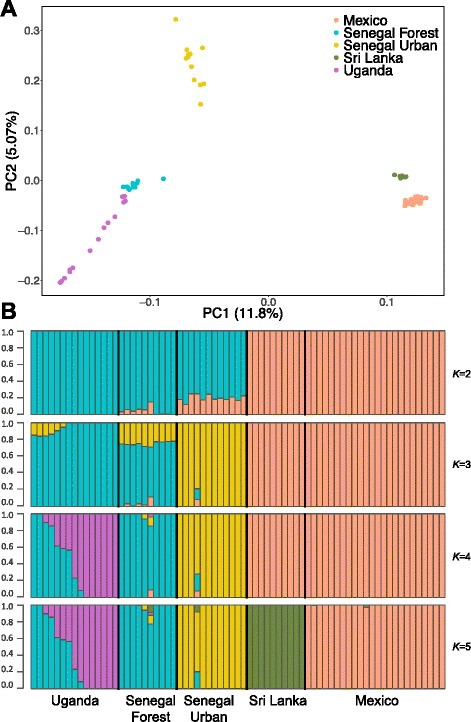
Genetic structure in *Ae. aegypti* populations. **a** Principal component analysis of *Ae. aegypti* exome sequences from five populations. The PCA was calculated from a covariance matrix calculated from all variants in the genome while accounting for genotype uncertainty. The percentage of the variance explained by each component is shown on the axis. **b** Ancestry proportions for *Ae. aegypti* individuals from five populations. Ancestry is conditional on the number of genetic clusters (*K* = 2–5) and is inferred from all sites in our dataset

There is clear genetic structure among the five populations we studied, with principal component analysis (PCA) clustering samples from the same location together. This analysis revealed three major groups in our data: Mexico + Sri Lanka, Uganda + Senegal Forest and Senegal Urban (Fig. 2a). Therefore, the Senegal Forest population is grouping with the population in Uganda rather than with the nearby Senegal Urban population.

This division between the Senegal Urban population and other populations in Africa is also apparent when an admixture analysis is used to infer the ancestry of the individuals from the five populations [35]. When we assumed that there were three ancestral populations (*K* = 3, Fig. 2b), the populations again grouped into Mexico + Sri Lanka, Uganda + Senegal Forest and Senegal Urban. Allowing higher levels of *K* recovers the division between Mexico and Sri Lanka and the genetic structure within the Ugandan population (Fig. 2b).

**Fig. 3:**
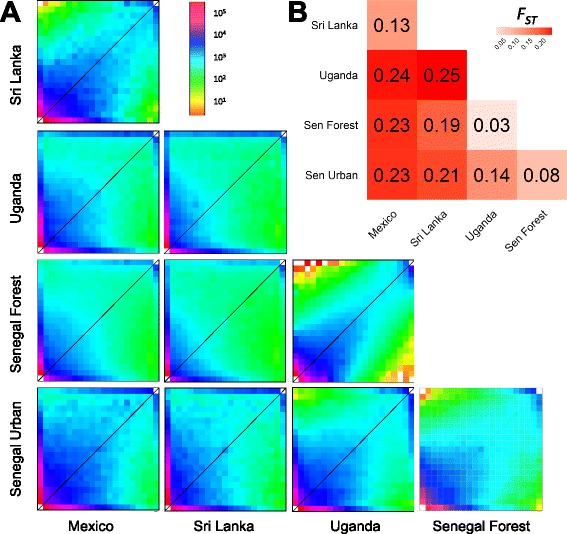
Differences in allele frequencies between populations. **a** Two-dimensional site frequency spectra. *Colours* represent the number of sites at a given frequency within each population (0-20) with frequency increasing from left to right and bottom to top in each spectrum. Allele frequencies were estimated using 10 randomly sampled individuals from each population. **b** Pairwise *F*_*ST*_

These patterns of population structure were broadly supported when we compared allele frequencies between populations using 2D site frequency spectra (SFS). Strikingly, the allele frequencies were markedly more similar when Senegal Forest was compared to Uganda than when it was compared to the relatively nearby Senegal Urban population (Fig. 3a). This is reflected in *F*_*ST*_ *,* which was greater between Senegal Urban and Senegal Forest (Fig. 3b; *F*_*ST*_ = 0.08) than between Uganda and Senegal Forest (*F*_*ST*_ = 0.03). Therefore, genetic differentiation between our African populations does not reflect geographic distance, but the Senegal Urban population is distinct from the other African populations. This is consistent with this population morphologically resembling the *Ae. aegypti aegypti* subspecies.

The frequency of alleles was strongly correlated in Sri Lanka versus Mexico (Fig. 3a), and *F*_*ST*_ between these populations was low (Fig. 3b). This supports a single out-of-Africa migration giving rise to these two populations. The non-African populations are clearly distinct from the African ones (Fig. 3; *F*_*ST*_ > 0.19 and different 2D SFS). Strikingly, the 2D SFS suggest that the Senegal Urban population is intermediate between the other African and the non-African populations (Fig. 3a). When Sri Lanka and Mexico are compared to Senegal Urban, there are more intermediate frequency polymorphisms in common than when these populations are compared to the other two African populations (Fig. 3a).

In Senegalese populations of *Ae. aegypti* there is evidence of polymorphic chromosomal inversions [52]. These are expected to suppress recombination and may lead to elevated differentiation between populations or species in these regions of the genome. This might be especially important around the sex-determining locus (sex in *Ae. aegypti* is determined by a single locus on an autosome) [52]. To examine this, we performed the principal component and admixture analyses on the three chromosomes separately and plotted *F*_*ST*_ in a sliding window across the genome. Although there appears to be some variation across chromosomes, we found no evidence that the patterns we see are driven by a single region of the genome or a single chromosome (Additional file 4).

### Domestic populations of *Ae. aegypti* in Senegal and outside of Africa share a different common ancestor from other African populations

Understanding the historical relationships between populations based on approaches like PCA, *F* statistics or admixture analysis is not straightforward [37, 53]. For example, the main groups distinguished by PCA are African versus non-African populations. PCA reflects the average coalescent times between pairs of samples [54], so this clustering may result from a bottleneck that occurred during the out-of-Africa migration rather than all the African populations sharing a different common ancestor from the non-African populations.

**Fig. 4:**
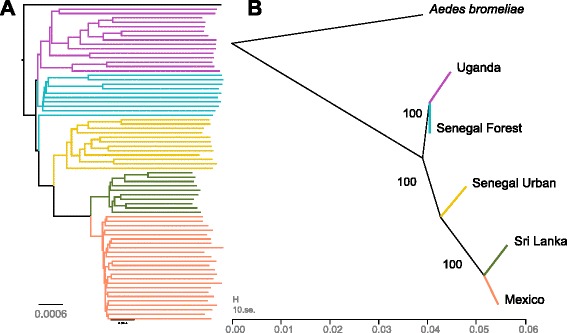
Historical relationships between *Ae. aegypti* populations. **a** Neighbour-joining tree of *Ae. aegypti* exome sequences from five populations. The tree is rooted with the sequence of *Ae. bromeliae*. Branches leading to samples from different populations are *colour-coded*. The scale is genetic distance (*D*_*xy*_). **b** Relationships between populations. The *branch lengths* are proportional to the amount of genetic drift that has occurred. The *scale bar* shows ten times the average standard error of the entries in the sample covariance matrix. The *numbers on branches* are percent bootstrap support calculated by resampling blocks of 100 SNPs. The population tree was reconstructed using allele frequency data using the TreeMix program [37]. Both panels use all sites in our dataset

To reconstruct historical relationships between the populations, we made rooted trees using *Ae. bromeliae* as an outgroup. The first approach we took was to draw a neighbour-joining tree based on the pairwise genetic distance (*D*_*xy*_) between our samples. With the exception of a single mosquito, the five populations formed five monophyletic groups (Fig. 4a). The major split within the tree separated Uganda + Senegal Forest from Sri Lanka + Mexico + Senegal Urban. Therefore, the pan-tropical *Ae. aegypti aegypti* populations shared a common ancestor with the population in Senegal that shares a similar ecology and has the classical phenotype associated with the *Ae. aegypti aegypti* subspecies.

To investigate these relationships further, we used allele frequency data to reconstruct the relationships among our populations (Fig. 4b). This again supported the hypothesis that among the populations sampled there has been a single ‘domestication’ of *Ae. aegypti* that presumably occurred in Africa, and this ancestral population has given rise to human-associated *Ae. aegypti* populations in Senegal, Asia and the Americas. This approach also estimates the amount of genetic drift that has occurred in these populations, which is a measure of their effective population size (branch lengths in Fig. 4b). From this it is clear that the effective population size of the Senegal Urban population has been reduced relative to *Ae. aegypti formosus* populations found elsewhere in Africa. There was a further increase in the rate of drift in the non-African populations, likely reflecting a bottleneck during the out-of-Africa migration.

Populations need not be related by a simple bifurcating tree, since they may also subsequently mix. An alternative hypothesis to explain the similarity of the Senegal Urban population to populations in Mexico and Sri Lanka is that *Ae. aegypti aegypti* from outside Africa have migrated back to Africa and mixed with the local *Ae. aegypti formosus* population [12]. This hypothesis has some support from the admixture analysis under the model that separates African and non-African populations (*K =* 2) with the Senegal Urban individuals all showing evidence of non-African ancestry (Fig. 2b; note this pattern is not seen at *K* > 2). We further tested whether the Senegal Urban population was a mixture of the nearby forest population and non-African populations using the three-population test of Reich et al. [38]. Regardless of whether we tested for admixture between Mexico or Sri Lanka and Senegal Forest, the *f3* statistic was positive, indicating that there was no evidence of admixture (source populations Senegal Forest and Mexico: *f3* = 0.008; source populations Senegal Forest and Sri Lanka: *f3* = 0.007). Furthermore, when we added migration events between the populations in Fig. 4b in the TreeMix model [37], we never detected any migration from outside Africa into Senegal Urban.

Despite finding no evidence using the three-population test for the Senegal Urban population being a mixture of African and non-African populations, we do find evidence for admixture among our five populations. We used the four-population test [38] to examine whether the allele frequencies were compatible with groups of four populations being related by a simple unrooted bifurcating tree without any mixing. We were able to reject this hypothesis in three cases ([[Mexico, Senegal Urban], [Senegal Forest, Uganda]]: *z* = –13.9, *p* < < 0.0001; [[Mexico, Sri Lanka], [Senegal Forest, Senegal Urban]]: *z* = –29.6, *p* < < 0.0001; [[Mexico, Sri Lanka], [Senegal Urban, Uganda]]: *z* = –27.2, *p* < < 0.0001). When we attempted to infer specific migrations between these populations using either *f3* statistics or TreeMix, we found that the results were inconsistent. Importantly, however, allowing migration does not alter the topology of the tree in Fig. 4b. Therefore, we can conclude that there has been some mixing between populations (possibly involving populations that we did not sample), but we are unable to infer which populations have mixed with each other.

### Domestic populations in Mexico and Senegal diverged very recently and experienced strong reductions in population size

**Fig. 5:**
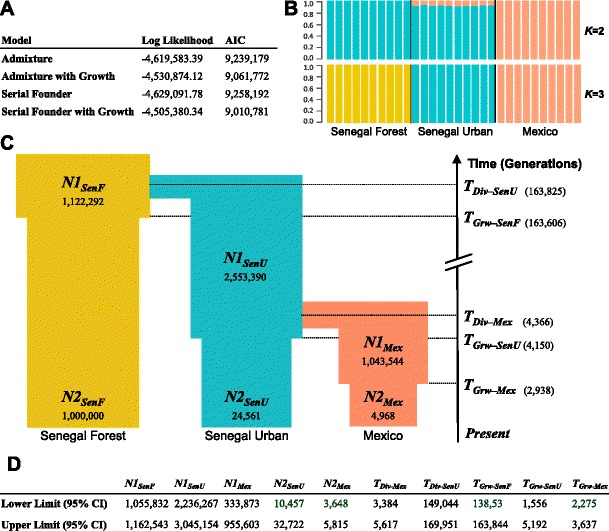
Demographic modelling for African and non-African populations does not support admixture-back-to-Africa model. **a** Statistical support for four demographic models. Log likelihood indicates likelihood of data given each model, with higher values corresponding to better fit. Lower Akaike information criterion (*AIC*) values indicate better support for model (AIC = 2*d* – 2(*Log Likelihood*), where *d* is the number of model parameters estimated). **b** Admixture analysis of data simulated under best-fit demographic model generates evidence for mixed ancestry in Senegal Urban similar to Fig. 2, despite including no admixture in model. Five thousand 500-bp exons were simulated using fastsimcoal2 and analysed using admixture [67]. **c** Schematic representing the maximum likelihood estimated model. Parameters are effective population sizes, and times when populations split or changed in size. **d** Confidence intervals (*CIs*) for model parameters

We next fitted explicit demographic models to our genetic data, both to provide an additional test of how our populations are related to each other, and to understand when population splits occurred and how population sizes changed [41]. We fitted two demographic models to pairwise 2D SFS from the Senegal Forest, Senegal Urban and Mexico populations (see Methods and Additional file 3). In the admixture-back-to-Africa model, Senegal Urban is admixed with non-African ancestry, while in the serial founder model Senegal Urban shares a common ancestor with non-African populations (Additional file 3). After extensive optimization of each model with and without population size changes, we found that a serial founder model with population size changes fit the data substantially better than any other model tested, with both a higher log likelihood (despite fewer parameters) and a considerably lower Akaike information criterion (AIC) value than the other models (Fig. 5a, Additional file 3). Therefore, modelling of demography supports the population relationships inferred above with an absence of gene flow back to Senegal Urban.

In apparent contradiction of these conclusions, our admixture analysis (Fig. 2b; *K* = 2) suggested that there may have been migration back to Senegal Urban from non-African populations. Similar results have been reported in previous admixture analyses of populations from Senegal [12]. However, changes in population size are known to create false signals of population mixing in such analyses [53]. To examine if this was the case here, we used our best-fit serial founder model (i.e. with no population mixing) to simulate sequence data. Repeating the admixture analysis on this simulated data, we found that Senegal Urban is assigned a similar level of mixed ancestry as we inferred from the real data (Fig. 5b versus Fig. 2b). Furthermore, this plot gives the incorrect impression that the two African populations are closely related (Fig. 2b). Therefore, our admixture analysis is compatible with the demographic model.

The demographic model allows us to infer when populations split and how their population size has changed (Fig. 5c, CIs in Fig. 5d). Following the split from the Senegal Urban lineage 4366 generations ago, the effective population size of the ‘Mexican’ lineage was initially large (~10^6^), suggesting that this ancestral population was still in Africa. Therefore, the two populations likely separated shortly before the out-of-Africa migration. Approximately 3000 generations ago, there was a strong reduction in the effective population size of the Mexican population, presumably reflecting a bottleneck associated with the out-of-Africa migration. Alongside this, the Senegal Urban population experienced a reduction in its effective population size ~4150 generations ago. The divergence of the Senegal Forest population from Senegal Urban and Mexico was considerably more ancient (163,825 generations ago).

### Adaptation during domestication

When the anthropophilic subspecies *Ae. aegypti aegypti* arose, it evolved a suite of characters that increased its capacity to vector dengue virus and yellow fever virus [10, 11, 14, 15]. Alongside this there were changes in colouration [14, 15], and the expansion into a novel ecological niche will likely have involved adaption to many other challenges. We examined sites that were strongly differentiated between the two subspecies, as these are likely to be enriched for sites that were selected in this transition. This is complicated, because the out-of-Africa migration was accompanied by large shifts in allele frequencies which are likely to obscure any effects of selection — we found 786 sites fixed for different alleles (*F*_*ST*_ = 1) in Senegal Forest versus Sri Lanka and 254 such sites when Senegal Forest was compared to Mexico (Additional file 5). By contrast there were just 3 such sites when Senegal Forest was compared to Senegal Urban (Additional file 5). Therefore, we focussed our analysis on the three African populations where the confounding effects of genetic drift are less strong. We scanned exomes from the three African populations using a normalised version of the population branch statistic (*PBSn1*) [55] to identify regions with strong differentiation specific to the Senegal Urban population. Our scan included 1,237,042 variable sites grouped into 240,609 non-overlapping windows of 5 SNPs spanning 13.17 Mb of the exome and nearby regions. We provide lists of strongly differentiated genes based on *PBSn1* and per-SNP *F*_*ST*_ in Additional files 5 and 6.

McBride et al. [5] found that odorant receptor 4 (*Or4*; AAEL015147) plays a key role in *Ae. aegypti aegypti’*s preference for feeding on humans. Three windows in our dataset tag this gene, but they show little evidence for genetic differentiation in any of the three domesticated populations (maximum windows: Senegal Urban *PBSn1* = 0.0701; Mexico *PBSn1* = 0.3135; Sri Lanka *PBSn1* = 0.2892). We similarly found no individual SNPs in this gene that were strongly differentiated between the subspecies (*F*_*ST*_; Additional file 5). Nonetheless, the 25 most differentiated genes included three odorant receptor/binding genes and a gustatory receptor (Table 1 and Additional file 6). Furthermore, the most differentiated gene encodes a pickpocket sodium channel, which is a family of proteins whose functions include olfaction and taste, and an ortholog (*ppk10*) of the gene we identified is associated with genetic variation in *Drosophila* olfaction [56]. While these are interesting candidates, to our knowledge, none of these genes have previously been implicated in habitat or host-seeking behaviour, nor were genes involved in taste or olfaction significantly overrepresented in this list relative to the genome average [57].

**Table 1:** Genes that are highly differentiated in the Senegal Urban population relative to Uganda and Senegal Forest

Gene	*PBSn1* ^a^	Location	Description
AAEL013219	0.713	Near_exon	Pickpocket sodium channel^b,c^
AAEL012960	0.685	Exon	Importin alpha^b^
AAEL010533	0.683	Intergenic	DNA binding^b^
AAEL014795	0.667	Intergenic	
AAEL001878	0.657	Exon	Lipase^b^
AAEL013222	0.652	Intergenic	Chitin binding^b^
*CYP12F7*	0.651	Intergenic	Cytochrome P450^b^
AAEL013025	0.649	Intergenic	
AAEL004516	0.648	Exon	Odorant binding protein [68]
*Gr19*	0.648	Exon	Gustatory receptor^b^
AAEL013637	0.640	Exon	Tyrosine catabolism^b^
AAEL008598	0.632	Intergenic	Lipid transport^b^
AAEL002764	0.632	Intergenic	Krebs cycle^b,c^
AAEL007277	0.628	Exon	tRNA editing^b,c^
AAEL007138	0.626	Near_exon	Sugar transporter^b^
*SCRBQ2*	0.624	Intergenic	Class B Scavenger Receptor^b^
AAEL001859	0.617	Exon	Vesicle transport^b^
AAEL004205	0.616	Intergenic	
AAEL000576	0.609	Exon	Lachesin^b^
AAEL010410	0.608	Intergenic	Odorant receptor^b^
AAEL001960	0.606	Intergenic	Cytochrome P450^b^
AAEL009058	0.605	Intergenic	
AAEL013215	0.605	Exon	Sulfonylurea receptor^b^
AAEL007345	0.605	Exon	Ion channel^b^
*Or50*	0.603	Exon	Odorant receptor^b^

^a^Normalised population branch statistic

^b^VectorBase gene description or Gene Ontology (*GO*) term

^c^FlyBase *Drosophila* ortholog

A key selection pressure on many *Ae. aegypti aegypti* populations is insecticides. An important mechanism involves changes to the target of DDT and pyrethroids that makes it insensitive to these insecticides (the voltage-gated sodium channel, aka *VGSC*, knock-down Resistance, *kdr*; AAEL006019) [58]. The gene encoding this protein is not exceptionally differentiated in this analysis — 53 windows fall within the coding region of *VGSC*, and we find only marginal evidence of differentiation (maximum windows: Senegal Urban *PBSn1* = 0.5457). However, two amino acid variants known to be associated with insecticide resistance are at frequencies of 73% and 85% in Mexico but absent elsewhere (Additional files 5 and 7; V756I and F1249C, which are referred to as V1016I and F1534C in previous annotations of the genome). Two genes in our top 25 encode two cytochrome P450s (*CYP12F7*, AAEL001960); cytochrome P450 is a family of proteins whose functions include breaking down insecticides in *Aedes aegypti* [59] (Table 1).

## Discussion

Using exome sequence data, we found that an urban population from Senegal was considerably more closely related to populations in Mexico and Sri Lanka than to a forest population just 420 km away. We estimate that the populations in urban Senegal and Mexico diverged just 4366 generations ago — 291 years ago if we assume 15 generations per year and a mutation rate of 3.5 × 10^–9^. By contrast, with the same assumptions, we estimate that the two nearby populations in Senegal split 10,921 years ago. The urban population in Senegal has the typical characteristics of the subspecies *Ae. aegypti aegypti* that is found throughout the tropics outside Africa: it lives alongside humans and has the characteristic pale scales on the first abdominal tergite [10, 14, 15]. Therefore, we can conclude that this population is a descendant of an ancestral African population of *Ae. aegypti aegypti* that evolved to be anthropophilic and subsequently colonised other continents, ultimately resulting in global pandemics of dengue virus, Zika virus and chikungunya virus.

Our conclusions contradict the prevailing model of *Ae. aegypti* evolution. Previous genetic studies have concluded that populations across sub-Saharan Africa are closely related and distinct from non-African populations (excluding some populations in coastal Kenya) [12]. Under this model, populations outside Africa belonged to the subspecies *Ae. aegypti aegypti,* while populations within Africa were *Ae. aegypti formosus.* Furthermore, anthropophilic populations in sub-Saharan Africa evolved independently from those outside Africa. Our data and analyses consistently reject this model.

An alternative scenario is that the urban population in Senegal arose when *Ae. aegypti aegypti* from elsewhere in the world migrated back to Africa. It is clear that this population is not directly derived from non-African populations, as it has greater genetic diversity than the Mexican or Sri Lankan populations (and this pattern has been consistently reported for other populations within and outside Africa [16]). Furthermore, the more plausible hypothesis that the Senegal Urban population was a mixture of African and non-African populations was rejected by three separate analyses: the formal test of admixture from Reich et al. [38], inferences of migration events in our population tree [37] and comparisons of explicit demographic models [41]. Therefore, we can conclude that the Senegal Urban population represents a close relative of an African population of *Aedes aegypti aegypti* that colonised other regions of the tropics.

Recent population bottlenecks result in a loss of rare genetic variants and reductions in genetic diversity. There was a considerably lower proportion of rare genetic variants in the *Ae. aegypti aegypti* populations from Senegal Urban, Mexico and Sri Lanka than in the *Ae. aegypti formosus* populations. Furthermore, genetic diversity was lowest outside of Africa, intermediate in the Senegal Urban population of *Ae. aegypti aegypti* and highest in the African *Ae. aegypti formosus* populations. This was reflected in the rates of genetic drift in these populations (Fig. 4b). Our demographic model confirmed that there was a sharp reduction in the effective population size during the out-of-Africa migration, presumably due to the small number of mosquitoes migrating out of Africa. Furthermore, genetic diversity is lower in Sri Lanka than in Mexico, which is consistent with other analyses that suggest that *Ae. aegypti* migrated to the New World first and subsequently colonised Asia [16, 17] (although a population bottleneck when this island was colonised from the mainland would produce the same pattern). Intensive control efforts may also have reduced population sizes and affected genetic diversity. However, the highest rate of genetic drift was in the common ancestor of the Sri Lankan and Mexican populations (Fig. 4b), suggesting that the reduction in the genetic diversity of these populations was due to a bottleneck caused by the out-of-Africa migration.

The sharp reduction in population size in the Mexican lineage (Fig. 5) allows us to estimate the date of the out-of-Africa migration as 2938 generations ago. Assuming 15 generations per year, this would be 196 years ago (95% CI: 152–242 years). The first historical record of the appearance of yellow fever in the New World that we are aware of was in 1648 [17], more than 100 years before our lower CI for the arrival of *Aedes aegypti*. Given that our estimates depend on the generation time of the mosquitoes and assumptions of our model such as the mutation rate, this small difference between genetic and historical data is expected.

The finding that close relatives of American and Asian *Ae. aegypti aegypti* exist side by side with *Ae. aegypti formosus* in Africa — and have remained genetically distinct — may have important implications for disease transmission. For example, *Ae. aegypti* is responsible for urban yellow fever outbreaks in West Africa but is not known to transmit the disease in East Africa [60], and it is tempting to speculate that this is due to the presence of *Ae. aegypti aegypti* being restricted to West Africa. Initial studies in Senegal indicated that *Ae. aegypti aegypti* populations have a substantially higher vector competence for dengue virus (DENV-2) than *Ae. aegypti formosus* [10], and similar results have been reported for yellow fever virus [11]. However, more work is needed, as this pattern was subsequently found not to hold when other virus genotypes were used [61]. In addition to high vector competence, *Ae. aegypti aegypti’s* importance as a disease vector results from it living alongside and biting humans [5]. It will be important to examine whether the genetic forms that we describe consistently differ in their ecology, behaviour and vector competence. For example, while our population of *Ae. aegypti formosus* in Senegal was from a forested area, our Ugandan population was from a human-disturbed region outside Kampala. Furthermore, previous studies in West Africa have found mosquitoes that morphologically resemble *Ae. aegypti formosus* breeding indoors [13]. Therefore, the extent to which *Ae. aegypti formosus* lives alongside and feeds on humans in Africa is unclear.

Another unanswered question is the distribution of the two forms across Africa. Further sampling and analysis will not only resolve this, but will also reveal the extent of gene flow between the two subspecies. This may help us understand why they have remained genetically distinct in Africa. In East Africa crosses have found no evidence of assortative mating or intrinsic reproductive incompatibilities [62]. However, a recent study in Senegal found that the two subspecies showed evidence of post-zygotic reproductive isolation [52]. It will also be of interest to understand how our populations are related to *Ae. aegypti aegypti* populations on the coast of Kenya which appear genetically distinct from other African populations [16].

Our results have important implications for the definition of the two subspecies of *Ae. aegypti.* The subspecies were originally defined based on colouration [7], but genetic studies have led many to view all populations in sub-Saharan Africa as *Ae. aegypti formosus* (excluding coastal Kenya; see Background). However, our results demonstrate that *Ae. aegypti aegypti* occurs in Senegal, and there is no conflict between genetic and morphological definitions of the subspecies in our dataset. Therefore, an important question is whether other African populations fall neatly into the two subspecies and whether they can be identified from morphological characteristics.

Why do our conclusions differ from those of previous studies? There have been numerous population genetics studies of *Ae. aegypti* in the past*,* most of which have used small numbers of genetic markers. Where datasets are small, there can be a lack of statistical power; for example, a previous study of 11 SNPs in Senegal found no significant genetic differentiation between the subspecies [10]. Many studies used mitochondrial DNA [63], but making inferences about the history of the entire genome from a single locus is problematic, with patterns inferred from mitochondrial DNA frequently differing from the nuclear genome [64–66]. Other studies have used microsatellites and the sequences of small numbers of nuclear loci and, more recently, larger datasets from RAD tag sequencing or SNP chips [12, 16, 19, 21].

In contrast to our results, previous studies of microsatellites and SNPs concluded that domestic *Ae. aegypti* populations in Africa arose separately from domestic populations elsewhere in the tropics [12, 16]. This conclusion was reached because African and non-African populations cluster separately in admixture and principal component analyses [12]. We see this same pattern (Fig. 2). However, drawing conclusions about the order of population splits from such analyses or from summary statistics like *F*_*ST*_ is not straightforward [37]. For example, principal component analysis is based on the average coalescent times between pairs of genomes, and this will be strongly affected by population bottlenecks [54]. Therefore, the reason that non-African populations do not cluster with *Ae. aegypti aegypti* from Senegal is not because these populations are unrelated, but is instead due to the population bottleneck associated with the out-of-Africa migration that caused large changes in allele frequencies that differentiate African from non-African populations. We confirmed this argument for our dataset by simulating data genomic data under our demographic model, and demonstrated that this led to distantly related African populations being incorrectly grouped together in an admixture analysis.

The genetic basis of the changes in vector competence and behaviour that occurred when *Ae. aegypti aegypti* evolved remains an important question. One approach to identify these changes is to look for regions of the genome that are strongly differentiated between the subspecies. This is greatly helped by comparing African populations of the two subspecies, as the shifts in allele frequencies that occurred during the out-of-Africa migration are likely to have obscured any effects of natural selection. We have catalogued the most strongly differentiated genes between subspecies in our dataset, and we hope that this list of candidate genes will be of interest to researchers interested in specific traits. However, to conclusively identify the genetic basis of adaptation, it will be necessary to include more populations, sequence the genome outside the exome to allow more powerful tests of selection and ultimately link these differences to phenotypic changes.

## Conclusions

We conclude that a domestic population of *Ae. aegypti* in Senegal and domestic populations on other continents share a different common ancestor from other African populations. The most parsimonious explanation of this observation is that an ancestral population of *Ae. aegypti* evolved to specialise on humans in Africa, giving rise to the subspecies *Ae. aegypti aegypti.* The descendants of this population are still found in Africa today. The rest of the world was colonised when mosquitoes from this population migrated out of Africa. Non-African populations are genetically distinct from African ones due to the population bottleneck that accompanied this migration.

## Additional files

Additional file 1: Details of mosquito samples used in this study. (XLSX 12 kb)
Additional file 2: Genome coordinates of regions that the exome capture probes were designed to target. The file is in BED format. (BED 2005 kb)
Additional file 3: Demographic models fitted. (A) Schematics of the demographic models tested and respective parameters. (B) Maximum likelihood estimate parameter values from admixture and serial founder demographic models. (C) Comparison of observed and expected 2D site frequency spectra under the different demographic models. (PDF 890 kb)
Additional file 4: Genetic structure across different chromosomes and regions of the genome. (A) Mean *F*_*ST*_ values for 1000-bp non-overlapping windows for each population pairwise comparison. The *x*-axis represents a physical map (bp) made by arranging scaffolds along the genetic map with scaffolds mapping to the same genetic map position being ordered randomly. Scaffolds according to Juneja et al. [23]. All positions with less than 10 individuals in each population comparison were excluded. Only windows containing at least 10 SNPs were plotted. (B) Ancestry proportions for *Ae. aegypti* individuals from five populations calculated for each chromosome separately. (C) Principal component analysis of *Ae. aegypti* exome sequences from five populations calculated for each chromosome separately. The PCA was calculated from a covariance matrix calculated from all variants in the dataset and accounting for genotype uncertainty. The percentage of the variance explained by each component is shown on the top of the plot. Ancestry is conditional on the number of genetic clusters (*K* = 2–5). (PDF 10034 kb)
Additional file 5:
*F*_*ST*_ calculated per SNP from called genotypes for all pairs of populations. A SNP is included if it is in the top 1000 highest values in any pairwise comparison of populations. *F*_*ST*_ is only reported where it is in the top 1000 for that comparison. Positions with less than 10 individuals in each population were excluded from the top highest values. (XLSX 1209 kb)
Additional file 6: The most differentiated regions of the genome between *Ae. aegypti aegypti* populations and *Ae. aegypti formosus* populations based on the normalised population branch statistic. The three tabs show highly differentiated regions between Senegal Forest + Uganda and Senegal Urban, Mexico or Sri Lanka. Data are analysed in 5-bp non-overlapping windows. (XLSX 117 kb)
Additional file 7: Polymorphisms in the *kdr* gene. Note that the annotation of this gene has changed, so the numbering refers to the genome version used in this manuscript and is different from that of most published work on this gene. (XLSX 204 kb)
