# Supplementary material for: Population genomics reveals that an anthropophilic population of Aedes aegypti mosquitoes in West Africa recently gave rise to American and Asian populations of this major disease vector
Source: BMC Biol. 2017 Feb 28;15:16. doi: 10.1186/s12915-017-0351-0 (PMC5329927; doi:10.1186/s12915-017-0351-0)

A Schematics of the demographic models tested and respective parameters. Note that in the Admixture-back-to-Africa model it is possible for $T_{div\text{-}Mex}$ to be greater than $T_{div\text{-}SenU}$, which alters the topology so the two Senegal populations are most closely related.

Serial Founder

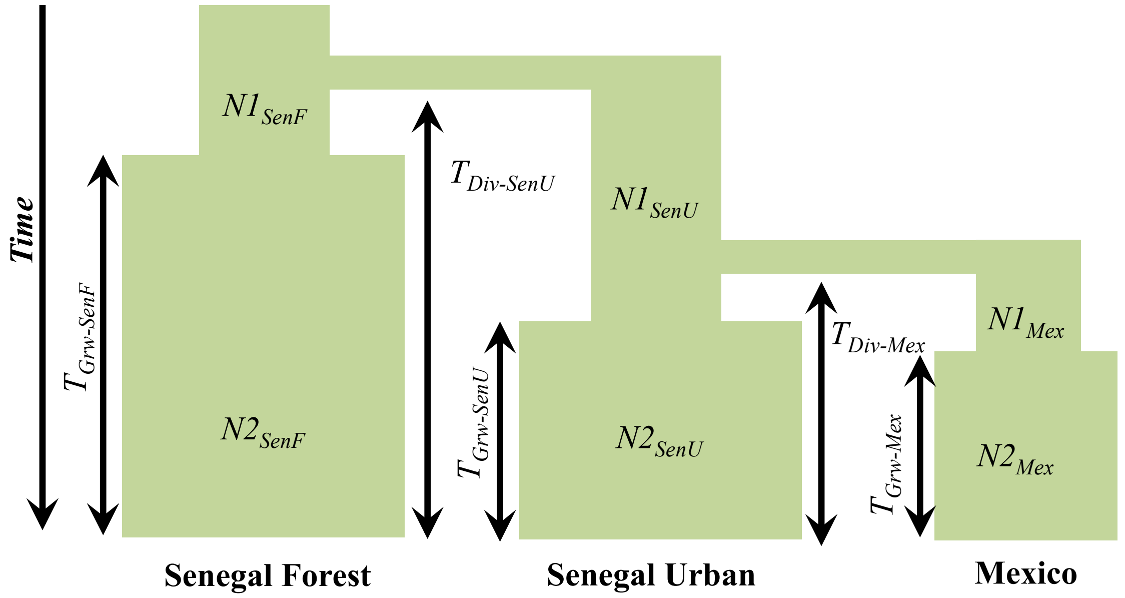

Admixture-back-to-Africa

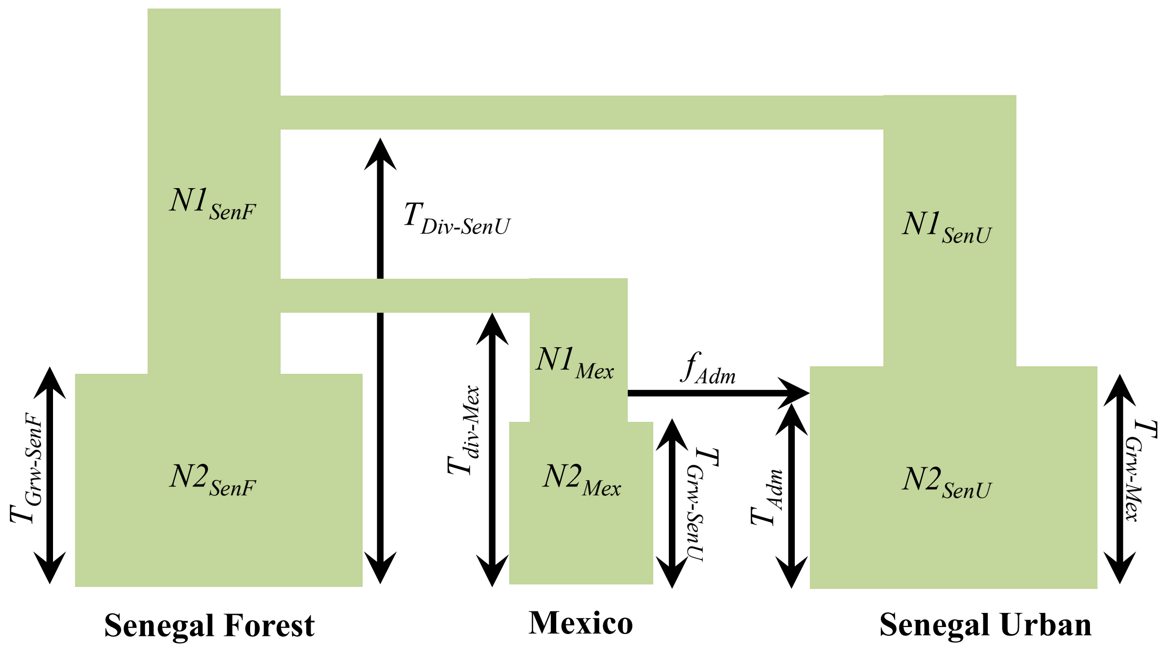

B Maximum Likelihood Estimate parameter values from Admixture and Serial-Founder demographic models.

Parameter	Admixture	Admixture with Growth	Serial Founder	Serial Founder with Growth (95% CI)
Log Likelihood	-4,619,583.39	-4,530,874.12	-4,629,091.78	-4,505,380.34
AIC^{A}	9,239,179	9,061,772	9,258,192	9,010,781
$N1_{SenF}$		1,268,992		1,122,292 (1,055,832-1,162,543)
$N1_{SenU}$		1,019,454		2,553,390 (2,236,267-3,045,154)
$N1_{Mex}$		543		1,043,544 (333,873-955,603)
$N2_{SenU}$	1,215,256	4,938	1,634,990	24,561 (10,457-32,722)
$N2_{Mex}^{B}$	4,310	6,085	9,495	4,968 (3,648-5,815)
$T_{Div-Mex}^{C}$	8,907	4,595	5,341	4,366 (3384-5617)
$T_{Div-SenU}$	215,878	98,576	173,350	163,825 (149,044-169,951)
$T_{Grw-SenF}$		587		163,606 (138,53-163,844)
$T_{Grw-SenU}$		81,362		4,150 (1,556-5,192)
$T_{Grw-Mex}$		1,365		2,938 (2275-3637)
T_{Adm}	2,141	1,434		
f_{Adm}^{D}	0.6507	0.5276		

A - Akaike Information Criterion (*AIC*) = 2*d* – 2(*Log Likelihood*), where d is the number of free variables in the model. We note that our *AIC* values are based on composite likelihood values and should therefore be interpreted with caution.
B - Effective sizes given in the number of diploid individuals. The current effective size of Senegal Forest was fixed as 10^6 in each model to reduce free parameters.
C - All time parameters (*T*) given in generations before present and are dependent on our assumption of a mutation rate of $3.5x10^{-9}$.
D – The fraction of Mexico lineages included in a single pulse admixture event within the Admixture models.

C Comparison of observed and expected 2D Site Frequency Spectra under four Maximum Likelihood Estimated demographic models. Each model was fit using Fastsimcoal2 (see Methods) and MLE models were plotted using *dadi*. Colors indicate the number of mutations in each bin as indicated by corresponding scales.

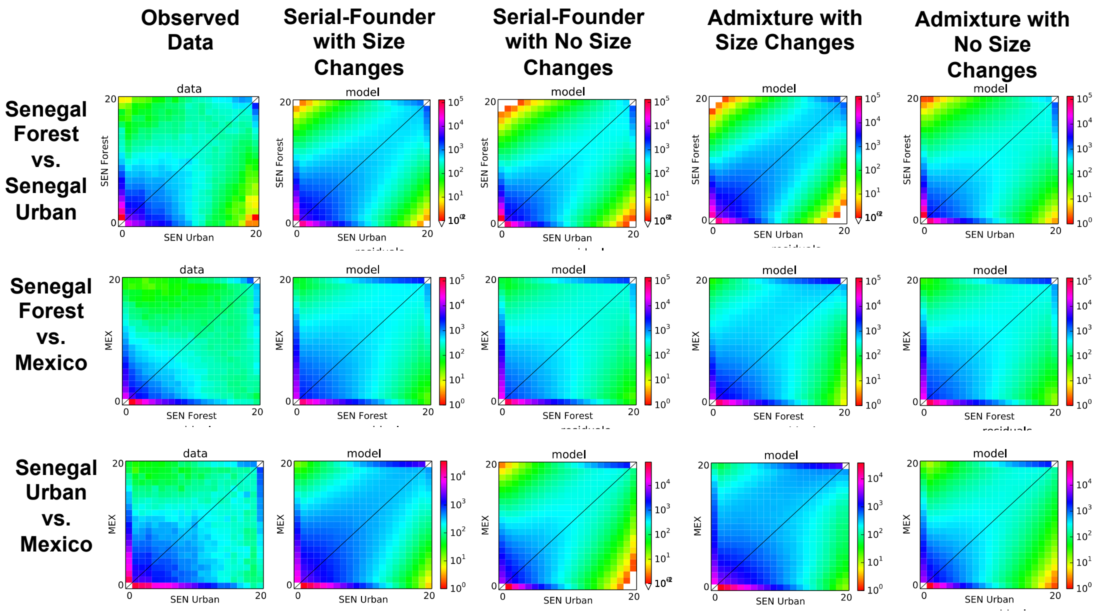

Supplement: Additional file 3: — Demographic models fitted. (A) Schematics of the demographic models tested and respective parameters. (B) Maximum likelihood estimate parameter values from admixture and serial founder demographic models. (C) Comparison of observed and expected 2D site frequency spectra under the different demographic models. (PDF 890 kb) [file 12915_2017_351_MOESM3_ESM.pdf]
