# Supplementary material for: Population genomics reveals that an anthropophilic population of Aedes aegypti mosquitoes in West Africa recently gave rise to American and Asian populations of this major disease vector
Source: BMC Biol. 2017 Feb 28;15:16. doi: 10.1186/s12915-017-0351-0 (PMC5329927; doi:10.1186/s12915-017-0351-0)
Supplement: Additional file 4: — Genetic structure across different chromosomes and regions of the genome. (A) Mean F ST values for 1000-bp non-overlapping windows for each population pairwise comparison. The x-axis represents a physical map (bp) made by arranging scaffolds along the genetic map with scaffolds mapping to the same genetic map position being ordered randomly. Scaffolds according to Juneja et al. [23]. All positions with less than 10 individuals in each population comparison were excluded. Only windows containing at least 10 SNPs were plotted. (B) Ancestry proportions for Ae. aegypti individuals from five populations calculated for each chromosome separately. (C) Principal component analysis of Ae. aegypti exome sequences from five populations calculated for each chromosome separately. The PCA was calculated from a covariance matrix calculated from all variants in the dataset and accounting for genotype uncertainty. The percentage of the variance explained by each component is shown on the top of the plot. Ancestry is conditional on the number of genetic clusters (K = 2–5). (PDF 10034 kb) [file 12915_2017_351_MOESM4_ESM.pdf]

A - Mean F_{ST} values for1000bp non-overlapping windows for each population pairwise comparison.

The x-axis represents a physical map (bp) made by arranging scaffolds along the genetic map with scaffolds mapping to the same genetic map position being ordered randomly. Scaffolds according to Juneja et al (2015 PLoS Pathogens. 11: e1004765). All positions with less than 10 individuals in each population comparison were excluded. Only windows contained at least 10 SNPs were plotted.

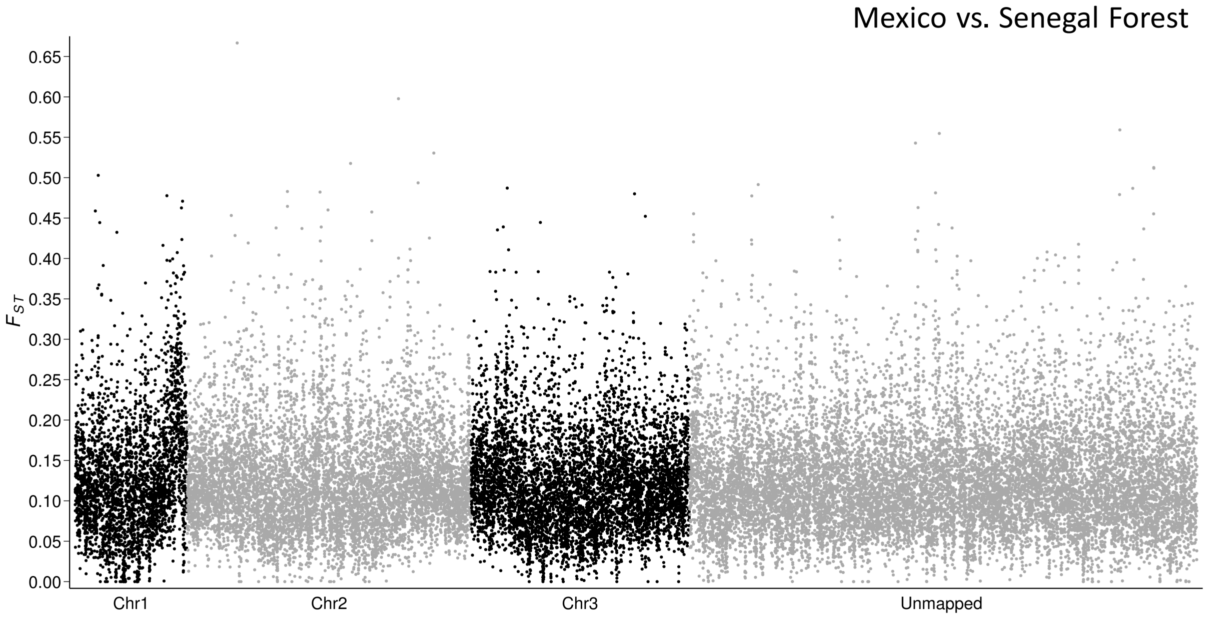

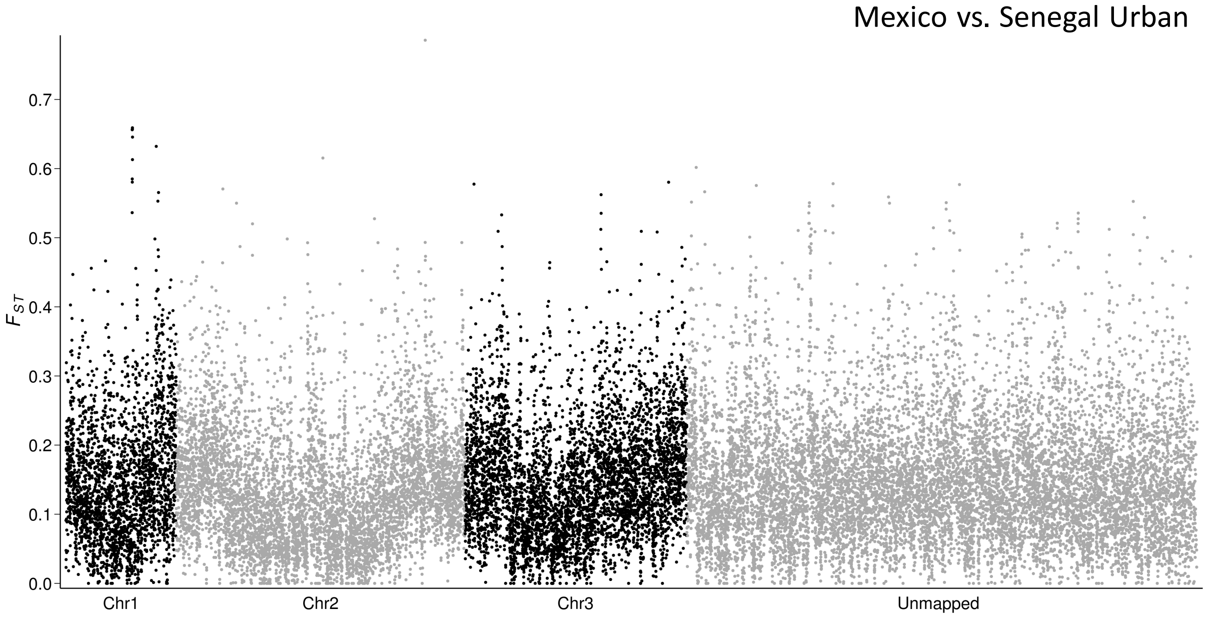

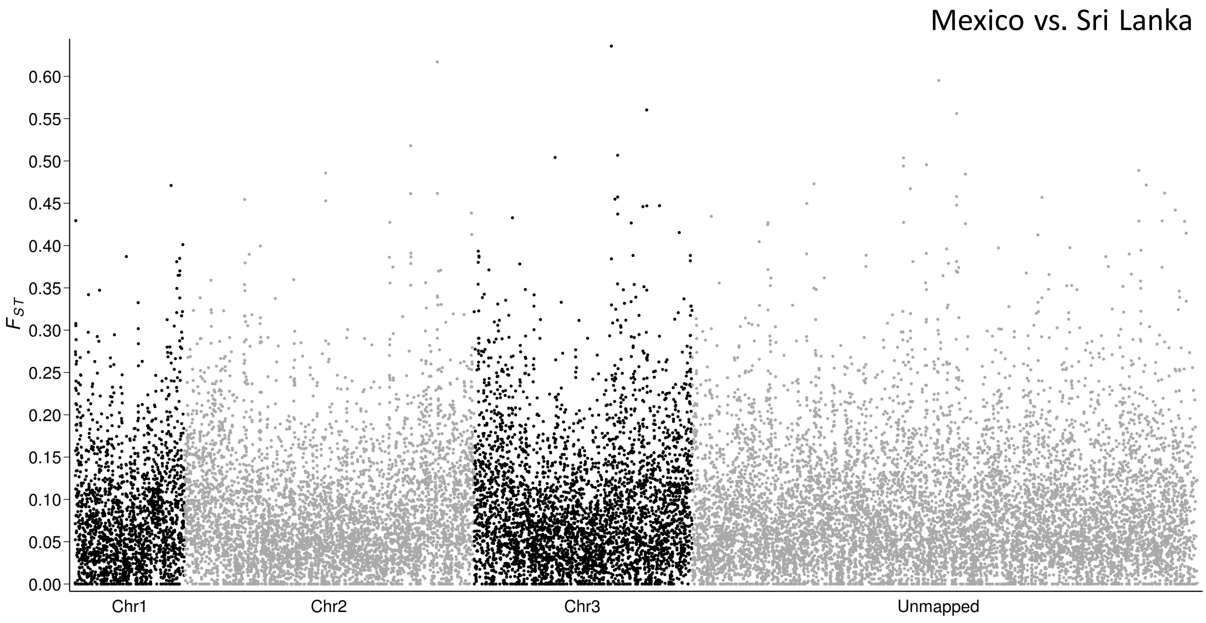
Mexico vs. Sri Lanka
F_{ST}
0.00
0.05
0.10
0.15
0.20
0.25
0.30
0.35
0.40
0.45
0.50
0.55
0.60
Chr1
Chr2
Chr3
Unmapped

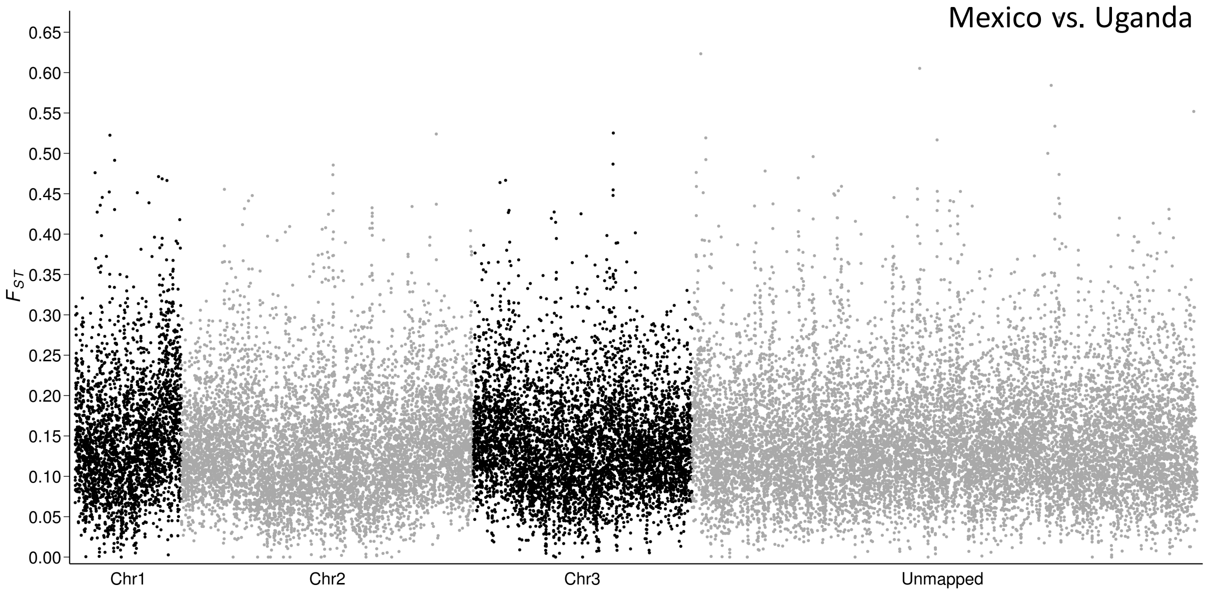
Mexico vs. Uganda
F_{ST}
0.00
0.05
0.10
0.15
0.20
0.25
0.30
0.35
0.40
0.45
0.50
0.55
0.60
0.65
Chr1
Chr2
Chr3
Unmapped

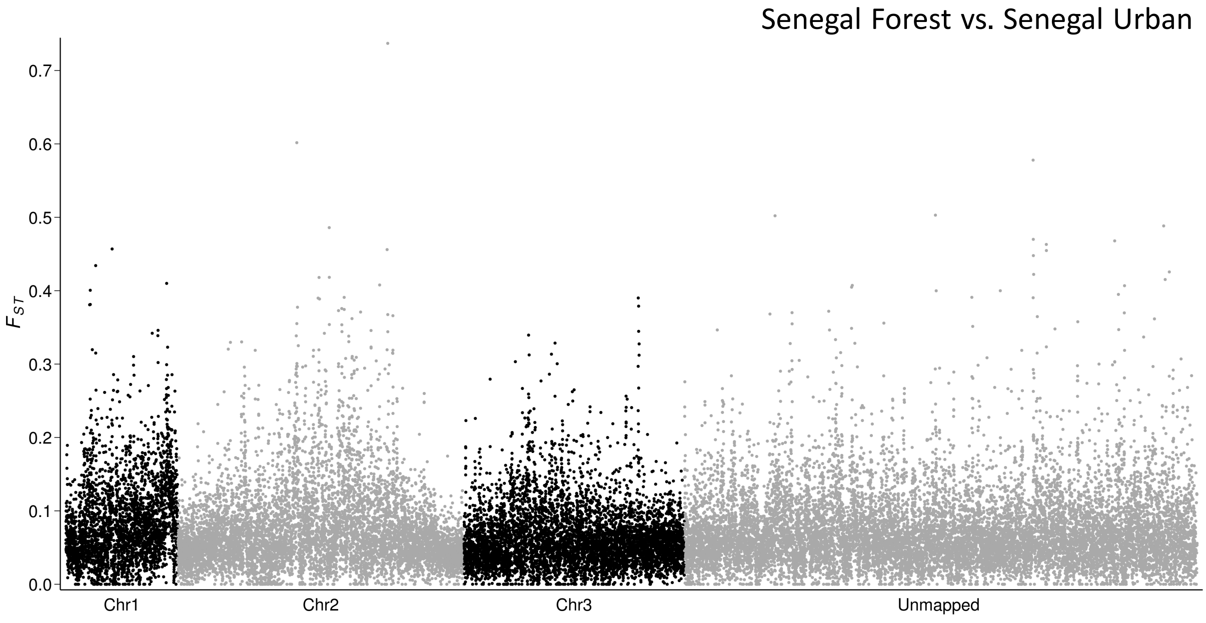
Senegal Forest vs. Senegal Urban
F_{ST}
0.0
0.1
0.2
0.3
0.4
0.5
0.6
0.7
Chr1
Chr2
Chr3
Unmapped

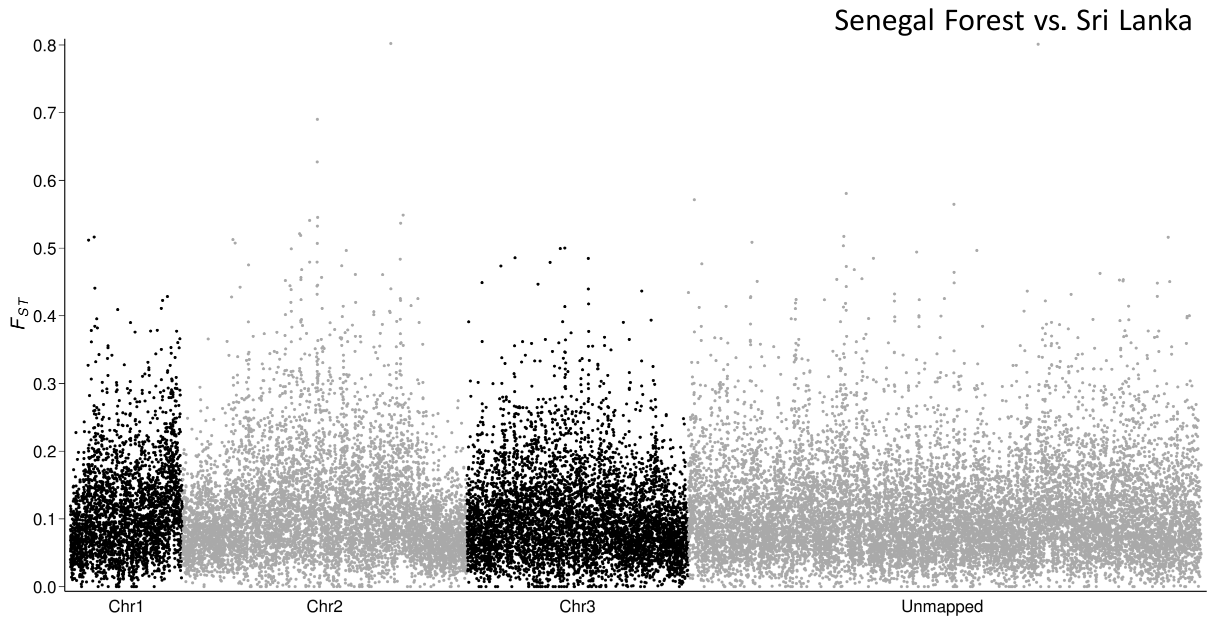
Senegal Forest vs. Sri Lanka
F_{ST}
0.0
0.1
0.2
0.3
0.4
0.5
0.6
0.7
0.8
Chr1
Chr2
Chr3
Unmapped

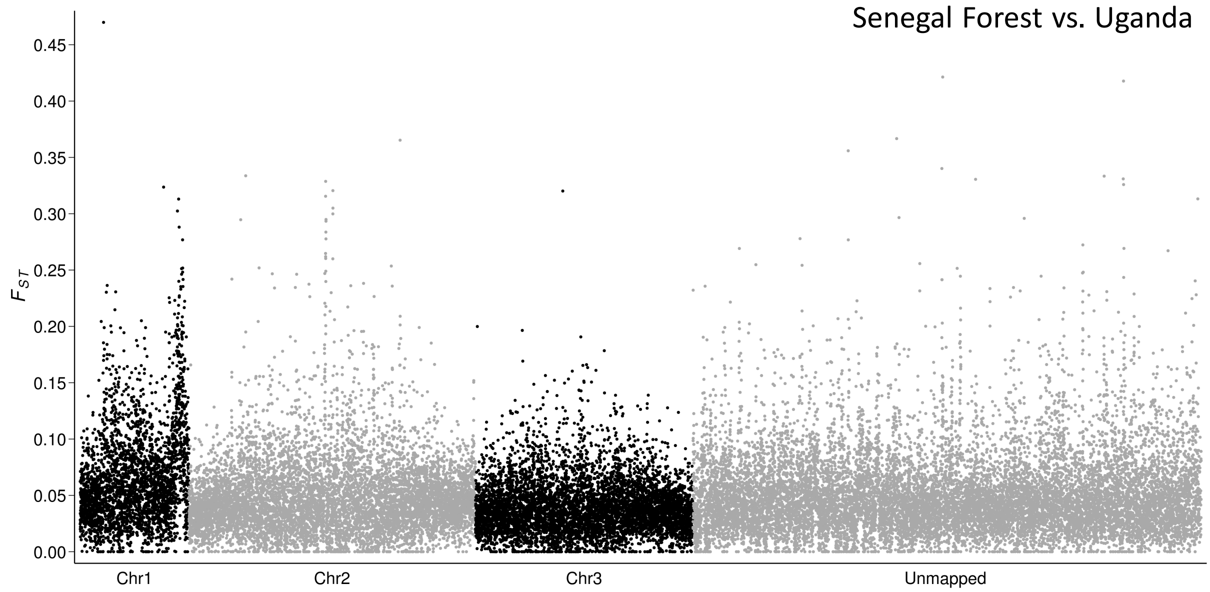
Senegal Forest vs. Uganda
F_{ST}
0.00
0.05
0.10
0.15
0.20
0.25
0.30
0.35
0.40
0.45
Chr1
Chr2
Chr3
Unmapped

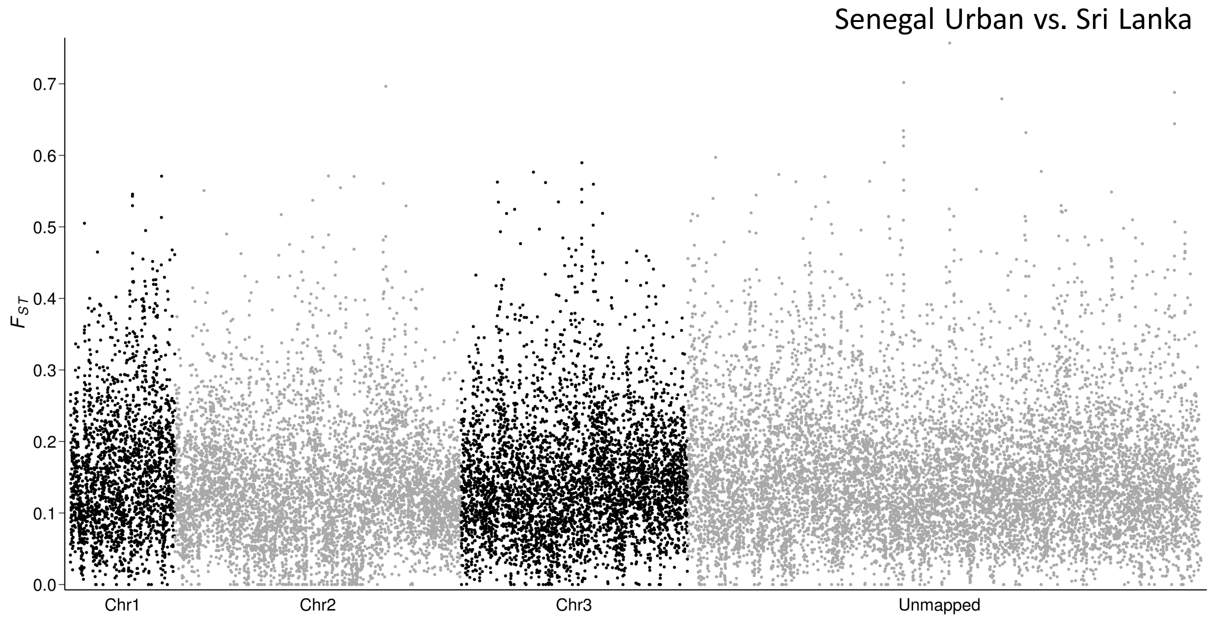
Senegal Urban vs. Sri Lanka
F_{ST}
0.0
0.1
0.2
0.3
0.4
0.5
0.6
0.7
Chr1
Chr2
Chr3
Unmapped

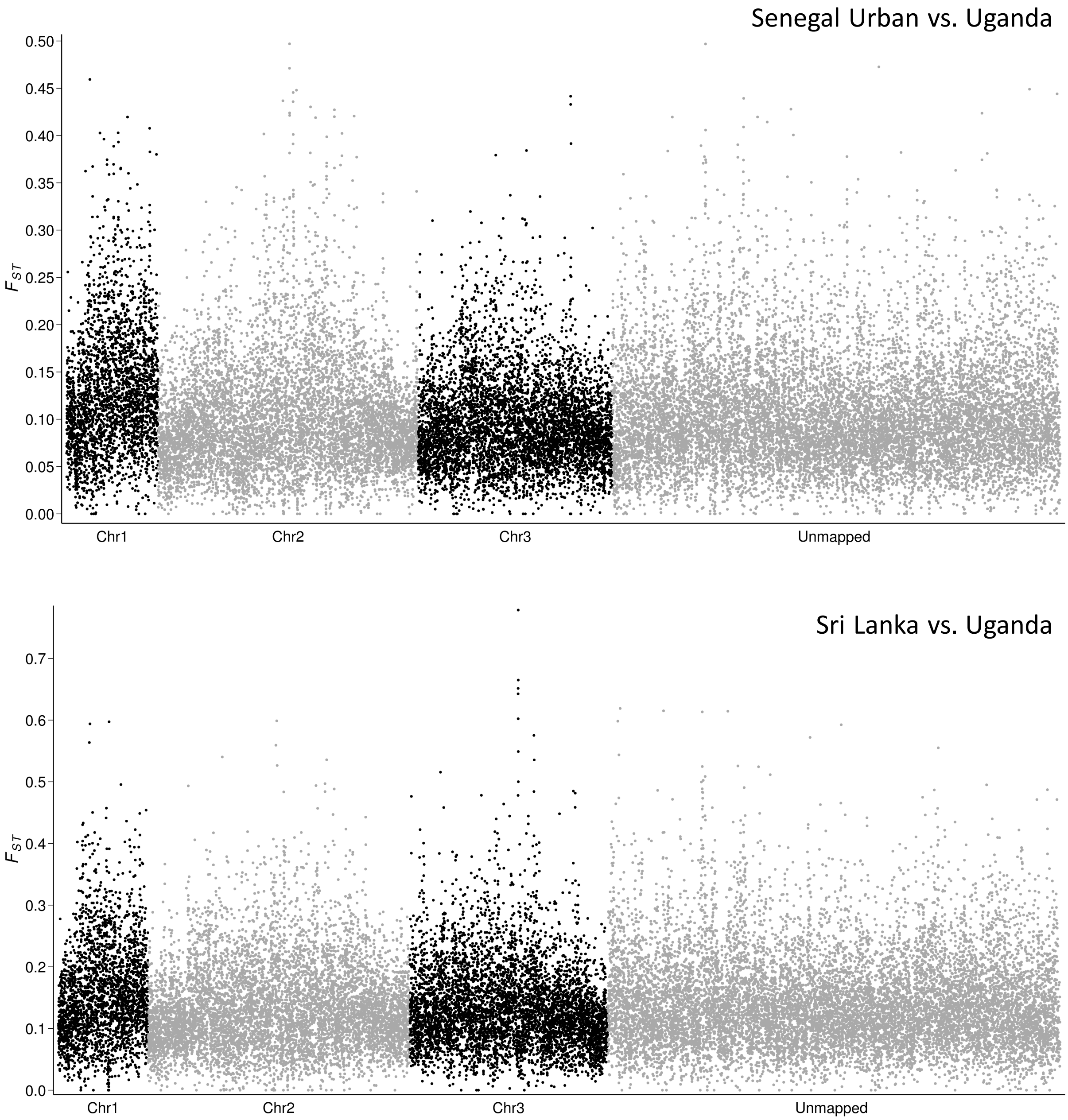
Senegal Urban vs. Uganda
0.50
0.45
0.40
0.35
0.30
0.25
0.20
0.15
0.10
0.05
0.00
F_{ST}
Chr1
Chr2
Chr3
Unmapped
Sri Lanka vs. Uganda
0.7
0.6
0.5
0.4
0.3
0.2
0.1
0.0
F_{ST}
Chr1
Chr2
Chr3
Unmapped

B - Ancestry proportions for *Ae. aegypti* individuals from five populations calculated for each of the chromosomes separately.

Ancestry is conditional on the number of genetic clusters (*K*=2–5), and is inferred from all sites in our dataset.

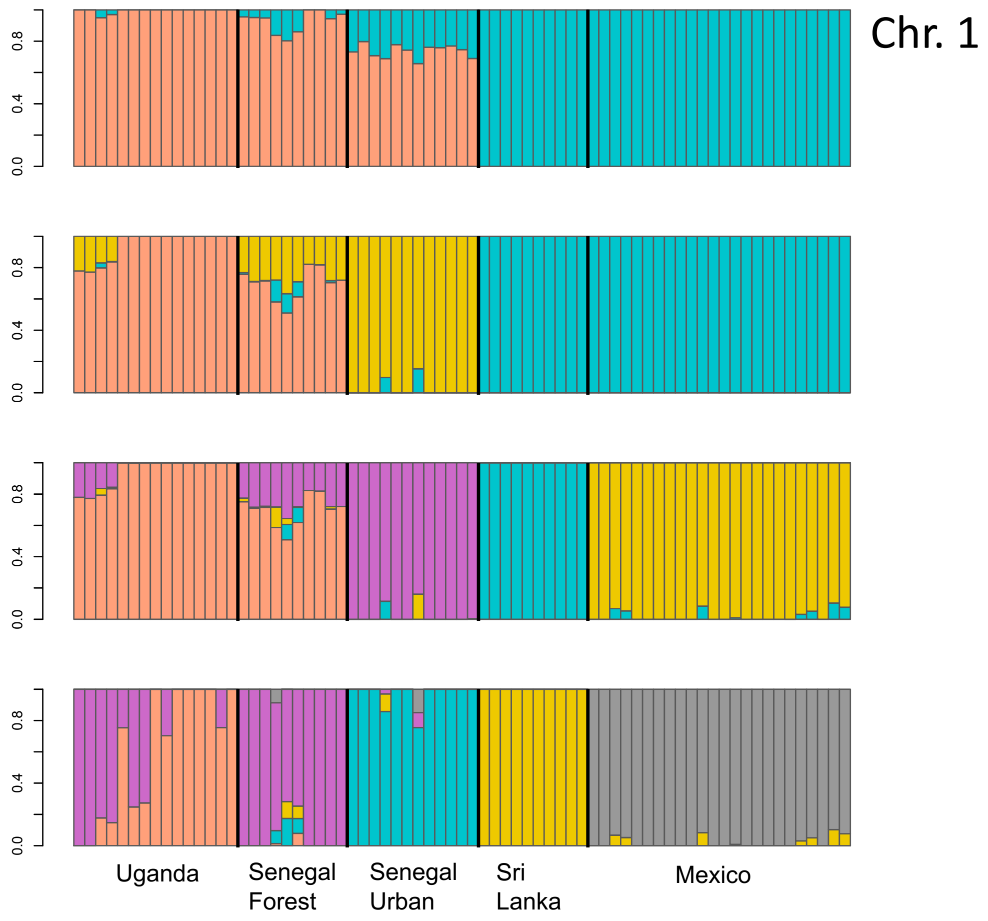

Chr. 2

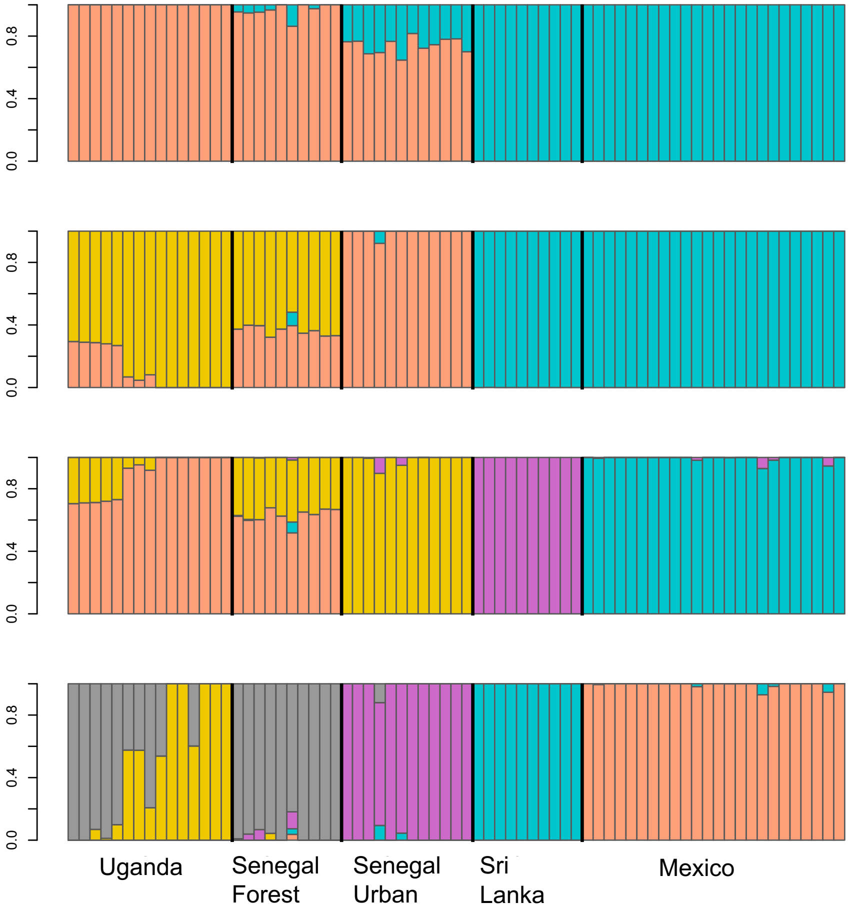
0.0
0.4
0.8
Uganda
Senegal Forest
Senegal Urban
Sri Lanka
Mexico

Chr. 3

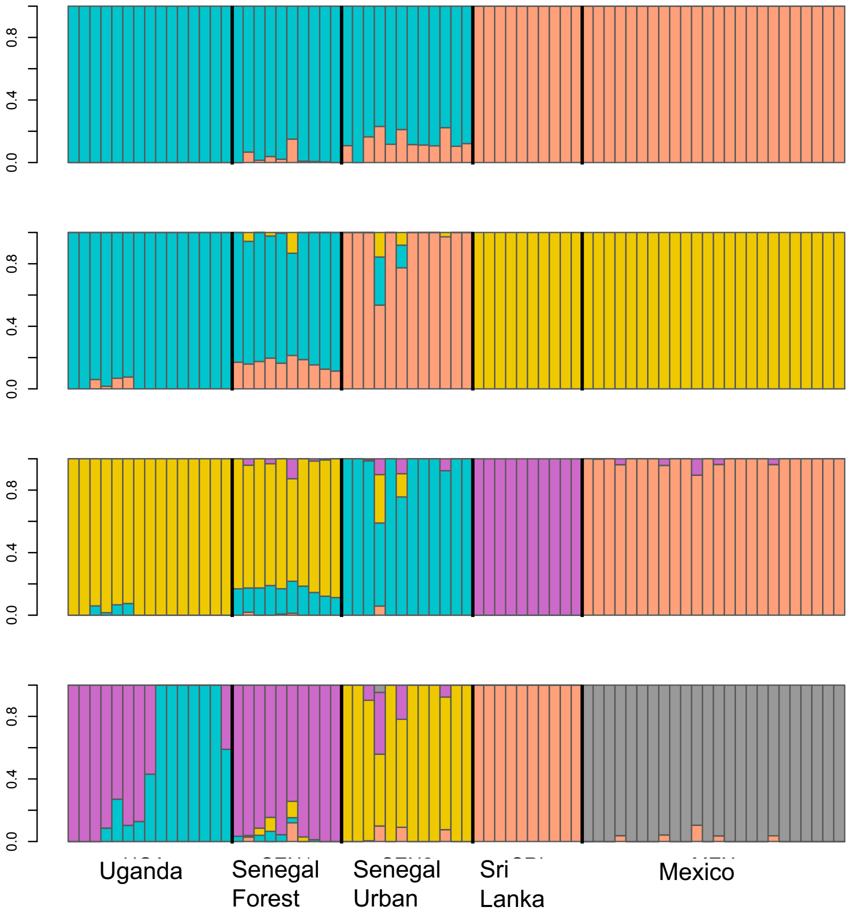
0.0
0.4
0.8
Uganda
Senegal Forest
Senegal Urban
Sri Lanka
Mexico

C - Principal components analysis of *Ae. aegypti* exome sequences from five populations calculated for each chromosome separately.

The PCA was calculated from a covariance matrix calculated from all variants in the dataset and accounting for genotype uncertainty. The percentage of the variance explained by each component is shown on the top of the plot.

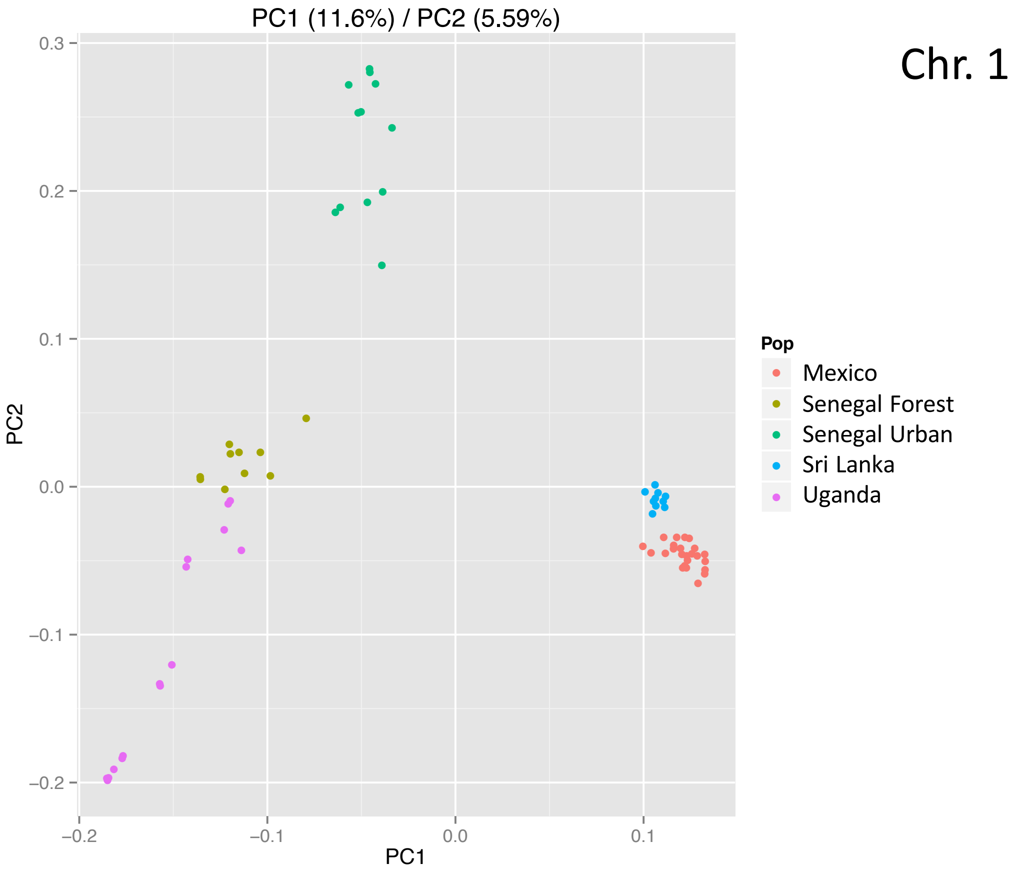

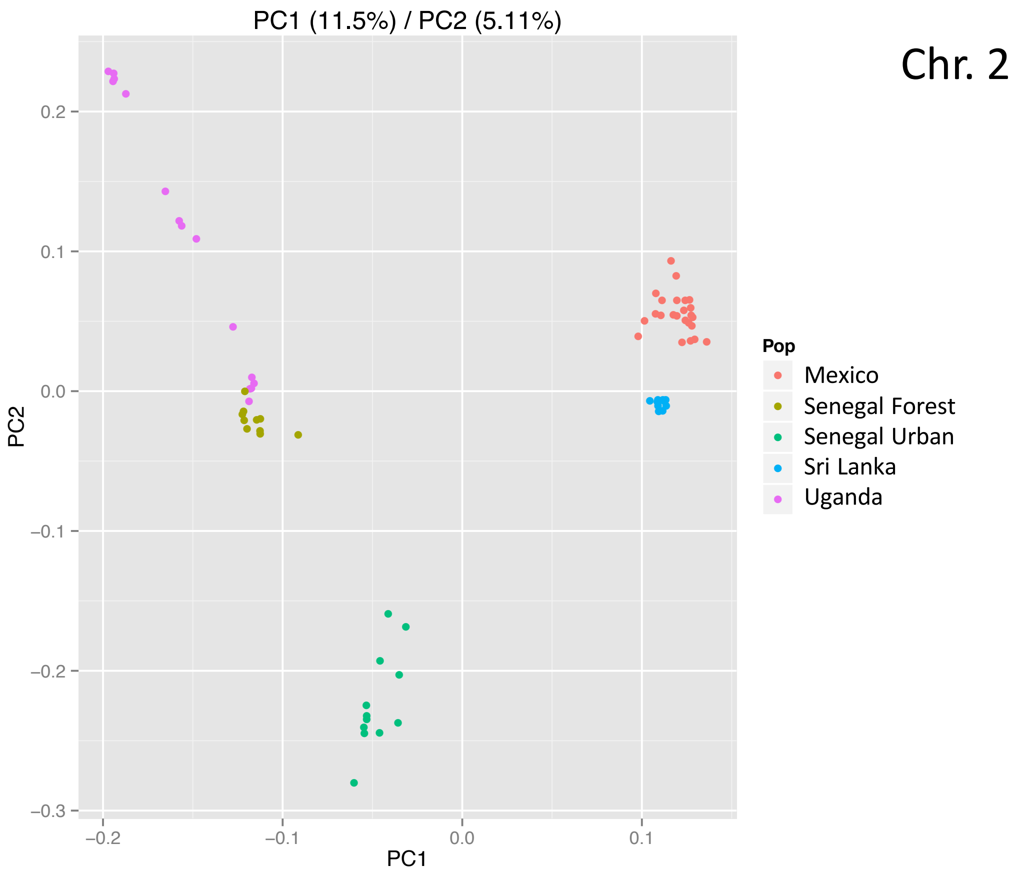

PC1 (11.5%) / PC2 (5.11%)
Chr. 2
PC2
PC1
0.2
0.1
0.0
−0.1
−0.2
−0.3
−0.2
−0.1
0.0
0.1
Pop
Mexico
Senegal Forest
Senegal Urban
Sri Lanka
Uganda

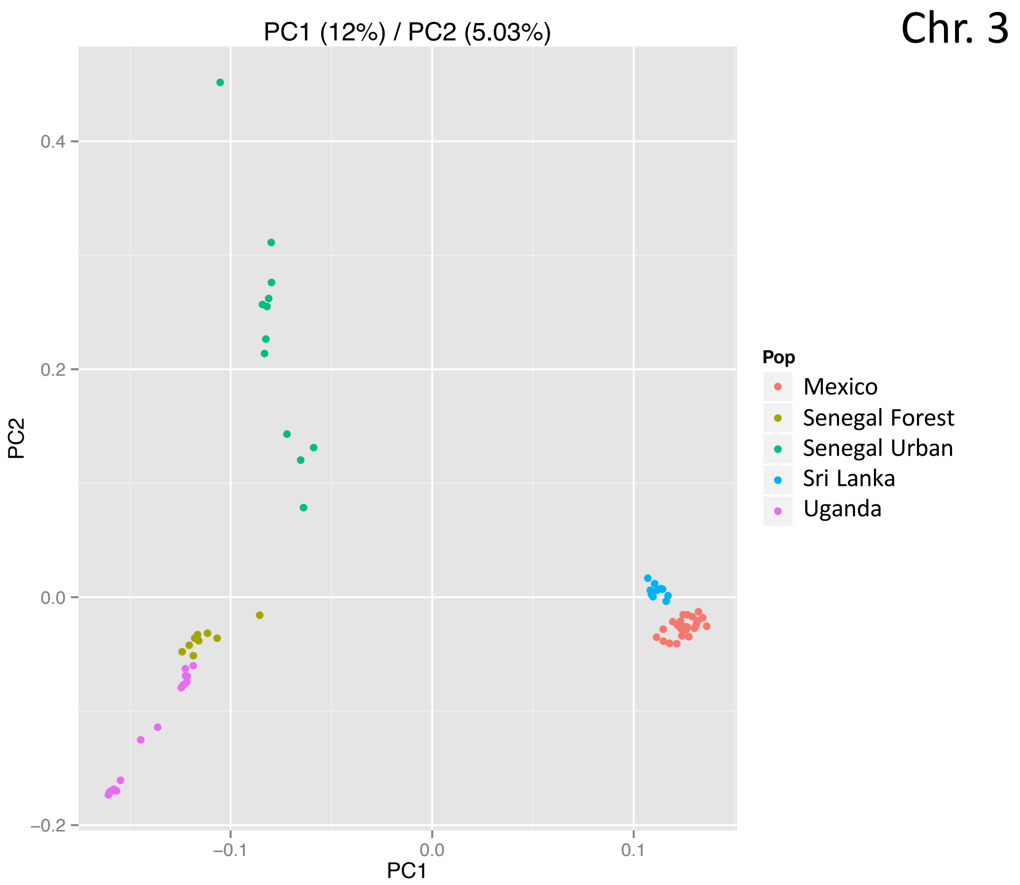

PC1 (12%) / PC2 (5.03%)
Chr. 3
PC2
PC1
0.4
0.2
0.0
−0.2
−0.1
0.0
0.1
Pop
Mexico
Senegal Forest
Senegal Urban
Sri Lanka
Uganda
